# Dual functional nanoplatforms potentiate osteosarcoma immunotherapy via microenvironment modulation

**DOI:** 10.1093/nsr/nwaf002

**Published:** 2025-01-10

**Authors:** Shunyi Lu, Yuqi Yang, Zhuorun Song, Jie Cao, Zhihui Han, Linfu Chen, Yunfei He, Jiayi Wang, Yun Teng, Zengli Zhang, Jun Zou, Jun Ge, Huilin Yang, Liang Cheng

**Affiliations:** Department of Orthopedic Surgery, The First Affiliated Hospital of Soochow University, Suzhou 215123, China; Institute of Functional Nano & Soft Materials (FUNSOM), Jiangsu Key Laboratory for Carbon-Based Functional Materials and Devices, Soochow University, Suzhou 215123, China; Institute of Functional Nano & Soft Materials (FUNSOM), Jiangsu Key Laboratory for Carbon-Based Functional Materials and Devices, Soochow University, Suzhou 215123, China; Department of Orthopedic Surgery, The First Affiliated Hospital of Soochow University, Suzhou 215123, China; Institute of Functional Nano & Soft Materials (FUNSOM), Jiangsu Key Laboratory for Carbon-Based Functional Materials and Devices, Soochow University, Suzhou 215123, China; Institute of Functional Nano & Soft Materials (FUNSOM), Jiangsu Key Laboratory for Carbon-Based Functional Materials and Devices, Soochow University, Suzhou 215123, China; Institute of Functional Nano & Soft Materials (FUNSOM), Jiangsu Key Laboratory for Carbon-Based Functional Materials and Devices, Soochow University, Suzhou 215123, China; Institute of Functional Nano & Soft Materials (FUNSOM), Jiangsu Key Laboratory for Carbon-Based Functional Materials and Devices, Soochow University, Suzhou 215123, China; Soochow University Institues for Translational Medicine, The First Affiliated Hospital of Soochow University, Suzhou 215123, China; Department of Orthopedic Surgery, Shanghai Jiao Tong University Affiliated Sixth People's Hospital, Shanghai 200233, China; Department of Orthopedic Surgery, The First Affiliated Hospital of Soochow University, Suzhou 215123, China; Department of Environmental Health School of Public Health, Soochow University, Suzhou 215123, China; Department of Orthopedic Surgery, The First Affiliated Hospital of Soochow University, Suzhou 215123, China; Department of Orthopedic Surgery, The First Affiliated Hospital of Soochow University, Suzhou 215123, China; Institute of Functional Nano & Soft Materials (FUNSOM), Jiangsu Key Laboratory for Carbon-Based Functional Materials and Devices, Soochow University, Suzhou 215123, China; Department of Orthopedic Surgery, The First Affiliated Hospital of Soochow University, Suzhou 215123, China; Institute of Functional Nano & Soft Materials (FUNSOM), Jiangsu Key Laboratory for Carbon-Based Functional Materials and Devices, Soochow University, Suzhou 215123, China

**Keywords:** osteosarcoma, metal nanoplatform, MnS_x_, H_2_S gas therapy, autophagy, immunotherapy

## Abstract

Osteosarcoma (OS), a highly aggressive bone tumor, presents significant challenges in terms of effective treatment. We identified that cellular autophagy was impaired within OS by comparing clinical OS samples through bioinformatic analyses and further validated the inhibition of mitochondrial autophagy in OS at the transcriptomic level. Based on this finding, we investigated the therapeutic potential of a dual functional metal nanoplatform (MnS_x_) to facilitate a transition from the protective effect of low-level autophagy in OS to the killing effect of high-level autophagy in OS. MnS_x_ facilitated intracellular H_2_S generation via endocytosis, leading to the S-sulfhydration of ubiquitin-specific peptidase 8 (USP8) and subsequent promotion of mitochondrial autophagy *in vitro*. Additionally, MnS_x_ activated the cyclic guanosine monophosphate–adenosine monophosphate synthase (cGAS)–stimulator of interferon genes (STING) pathway, further enhancing the cellular autophagic response and accelerating tumor cell death. Moreover, it was demonstrated *in vivo* that MnS_x_, on the one hand, mediated the activation of tumor autophagy by USP8 via intracellular H_2_S, while Mn^2+^ promoted the maturation of dendritic cells, activated cytotoxic T lymphocytes and contributed to tumor eradication. Such tumor killing could be suppressed by the autophagy inhibitor chloroquine. Importantly, synergistic combination therapy with immune checkpoint inhibitors showed promise for achieving complete remission of OS. This study highlights the potential of MnS_x_ as a dual-functional therapeutic platform for OS treatment and offers novel directions for future research in this field.

## Teaser

A dual functional metal nanoplatform (MnSx) was proved to facilitate a transition from the protective effect of low-level autophagy to the killing effect of high-level autophagy in osteosarcoma.

## INTRODUCTION

Osteosarcoma (OS), a highly aggressive form of bone cancer, predominantly affects adolescents and older adults. Despite being relatively rare, OS is characterized by its aggressive nature, frequent local recurrence and systemic progression [1], which contribute to poor long-term survival rates and pose substantial challenges for patient outcomes and quality of life. The conventional treatment regimen for OS includes a combination of surgery, chemotherapy and radiotherapy [2]. Neoadjuvant chemotherapy, introduced in the 1970s, has significantly enhanced the 5-year event-free survival rate of OS patients but the improvement in survival has stagnated over time [3]. A major hurdle in the treatment of OS is the inherent resistance of tumor cells to traditional chemotherapy agents, which is attributed to their genetic diversity and adaptability [4,5]. Additionally, the adverse effects associated with chemotherapy continue to significantly impact the well-being of OS patients.

Recent advances in oncoimmunology have shed light on the clinical trials focused on reprogramming the immune system to better recognize cancer cells [6,7]. Extensive characterization of immune infiltrates in OS and molecular profiling have revealed potential immune therapeutic targets [8,9]. Pre-clinical studies have shown promise in the use of immunotherapy for treating OS [10–12]. Nevertheless, obstacles persist, as OS frequently presents as an immunologically quiescent microenvironment with inadequate immune cell infiltration. However, the precise etiology of the immunologically quiescent microenvironment remains to be elucidated. Emerging research suggests that modifying the immune microenvironment in OS could be the key to effectively eliminating the tumor [13,14]. In recent years, nanomaterials have gained attention as innovative strategies in cancer immunotherapy due to their ability to deliver targeted therapies and interact with the immune system, thereby inducing or enhancing the anti-tumor immune response [15,16]. Notably, inorganic nanomaterials have shown significant potential in modulating the immune microenvironment of tumors [6,17]. Manganese (Mn), an essential trace mineral in the human body, plays a pivotal role in activating the host immune system via the cyclic guanosine monophosphate–adenosine monophosphate (GMP–AMP) synthase (cGAS)–stimulator of interferon genes (STING) pathway, enhancing anti-tumor immunotherapy [18]. Specifically, Mn ions have been demonstrated to stimulate dendritic cells (DCs) and macrophages, facilitating tumor-specific antigen presentation. They also activate cytotoxic T lymphocytes (CTLs, CD8^+^ T cells) and natural killer (NK) cells, which are crucial for the elimination of tumor cells [6,19]. Additionally, Mn^2+^ has been shown to inhibit immune checkpoint blockade (ICB), reducing the ability of tumors to evade the immune system [20].

Although manganese-based treatments have shown efficacy in other cancers, the unique challenges posed by the OS microenvironment, such as strong recurrence and extremely poor prognosis, may limit the effectiveness of these strategies. Autophagy is an intracellular degradation process that can eliminate and recycle damaged proteins and organelles, thereby prolonging cell lifespan. OS cells can increase their survival and proliferation through low levels of autophagy, induce immunosuppression and acquire resistance to chemotherapy [21,22]. However, a novel gas molecule, hydrogen sulfide (H_2_S), shows potential for effectively regulating cellular autophagy levels and treating tumors. As the third gasotransmitter, along with nitric oxide and carbon monoxide [23], H_2_S plays significant roles in various physiological and pathological conditions and has been shown to modulate cellular autophagy levels [24–26]. H_2_S has been linked to the inhibition of OS progression via the EGFR/PI3K/Akt signaling pathway, highlighting its connection to autophagic processes in OS [24].

**Scheme 1.**
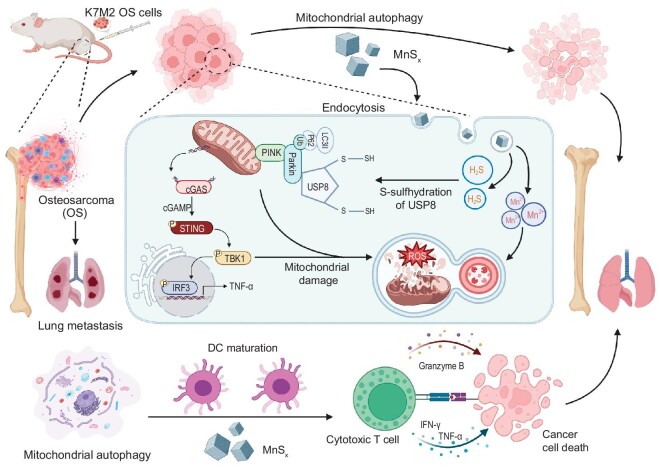
Dual functional nanoplatform-mediated mitochondrial autophagy enhances OS immunotherapy through microenvironmental modulation.

The present study proposes a novel manganese sulfide platform for OS treatment, which initially enhances autophagy in OS cells by releasing hydrogen sulfide gas. This process effectively disrupts the protective effect of low-level autophagy on OS, thereby achieving the killing effect of high-level autophagy on OS and inducing immunogenic cell death (Scheme 1). Following this, the platform adjusted the immune microenvironment of OS, with sequentially released manganese ions promoting DC maturation via the cGAS-STING pathway, enhancing antigen presentation and ultimately leading to tumor eradication through cellular immunity. This comprehensive approach holds promise for revolutionizing the treatment landscape for OS.

## RESULTS AND DISCUSSION

### Autophagy related to osteosarcoma

**Figure 1.**
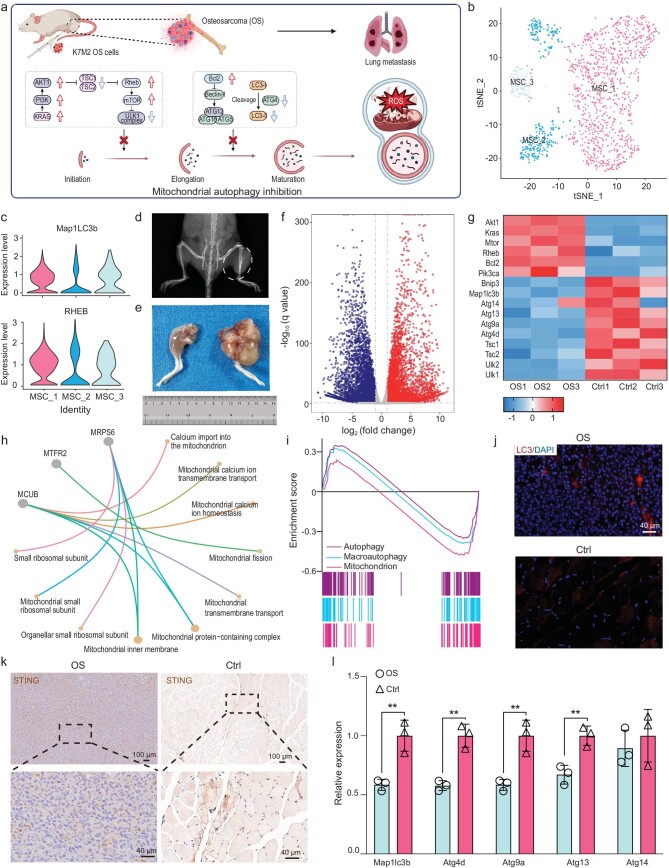
Autophagy inhibition in osteosarcoma. (a) Inhibition of mitochondrial autophagy within OS (created with BioRender.com). (b) t-SNE plots of MSCs identified in seven OS lesions and four healthy bone tissues, colored according to the three cell subclusters shown. (c) Violin plots showingnormalized expression levels of autophagy-associated genes for each cell cluster marker. (d) X-ray imaging of osteosarcoma *in situ* on Balb/c mice. (e) General view of osteosarcoma. (f) Volcano map of differentially expressed genes (DEGs) in osteosarcoma tissue (OS) and contralateral muscle tissue (Ctrl). (g) Heatmap summarizing the DEGs related to autophagy-related genes. (h) The GO biological process enrichment analysis of DEGs. (i) Gene set enrichment analyses for genes participating in autophagy. (j) Immunofluorescence staining of LC3 in OS and muscle tissue. (k) Immunohistochemical staining of STING in OS and muscle tissue. (l) RNA expression levels and quantitative results of autophagy-related genes in the OS and Ctrl groups. Statistical significance was calculated by one-way analysis of variance (ANOVA) with Tukey's *post hoc* test.

The autophagy pathway serves as a crucial protective mechanism within cells, guarding against damage from reactive oxygen species (ROS) to intracellular proteins and organelles [27]. Moreover, accumulating evidence underscores the necessity of a basal level of autophagy for normal organismal function [28]. We downloaded data from the Gene Expression Omnibus (GEO) for OS tissue and healthy tissue samples to analyze differentially expressed genes (DEGs) between the two tissues. Three cellular subclusters of mesenchymal stem cells (MSCs) were identified using the t-distributed stochastic neighbor embedding (t-SNE) method (Fig. 1b), representing MSC subclusters 1, 2 and 3, respectively. It was found that cellular subcluster 3 consisted of cells within healthy bone tissue, while cellular subclusters 1 and 2 were predominantly OS cells. We then analyzed the expression profiles of a set of characteristic genes in each MSC subcluster (Fig. 1c and Fig. S1), which included (i) subcluster 3, which was predominantly characterized by high expression of the autophagy-associated cellular markers MAP1LC3B (LC3B), PTEN and BNIP3, and (ii) subcluster 1, which was characterized by relatively high expression of RHEB. This finding suggested that autophagy signaling was suppressed in OS tissues, and that the protective mechanism of low-level autophagy contributes to the growth of OS, which is a significant factor in its highly aggressive nature.

To further validate the relationship between autophagy and OS, we established an OS model by *in situ* inoculation of Balb/c mouse tibia with K7M2 cells and monitored the progression of OS via X-ray imaging (Fig. 1d, white circle). After the mice were sacrificed, we conducted ribonucleic acid (RNA) transcriptome sequencing on OS tissues and healthy muscle tissues (Fig. 1e). DEGs in tumor tissue exhibited significant disparities compared to those in contralateral normal tissue (Figs 1f and S2). We further examined autophagy-related genes in both groups using a heatmap, and the results indicated a notable decrease in the expression of autophagy-related genes, especially LC3B, in the OS group, along with a conspicuous increase in autophagy-inhibiting genes, which was consistent with the results of single-cell RNA sequencing (Fig. 1g). Gene ontology (GO) analysis revealed mitochondrial dysfunction within the tumor tissues (Fig. 1h). Kyoto Encyclopedia of Genes and Genomes (KEGG) analysis revealed enrichment of dysfunctional lysosomes in tumor tissues (Fig. S3). Autophagy is divided into macroautophagy and mitochondrial autophagy; therefore, by enrichment analysis of the gene set enrichment analysis (GSEA) autophagy gene set, we found that autophagy was downregulated in OS tissues, while the leading-edge subset gene was more significantly differentially expressed in the gene set of mitochondrial autophagy-related genes (Fig. 1i). Consequently, it could be assumed that mitochondrial autophagy was impaired in OS tissues. Mitochondrial integrity and lysosomal function are crucially regulated by autophagic clearance, a specialized form of autophagy [29]. Additionally, immunofluorescence staining analysis revealed a reduction in the expression level of LC3 in OS tumor tissue relative to normal muscle tissue (Fig. 1j). Immunohistochemistry demonstrated decreased STING expression levels in OS (Fig. 1k). The expression of autophagy-related genes in OS tissues was further verified by reverse transcription-polymerase chain reaction (RT-PCR), which revealed that autophagy was suppressed in OS (Fig. 1l). Numerous studies have documented the ability of hydrogen sulfide (H_2_S) to promote autophagy, with exogenous H_2_S facilitating the clearance of damaged mitochondria [30,31]. The objective of this study was to investigate the potential use of H_2_S to modulate mitochondrial autophagy in OS, with a particular focus on shifting the balance from limiting immunogen release to promoting damage-associated molecular pattern (DAMP) release. This could have significant implications in the treatment of OS, where low levels of autophagy act as a protective barrier against the disease but high levels of autophagy could be harnessed to produce tumor-killing effects.

### Preparation and characterization of MnS_x_ nano particles

**Figure 2.**
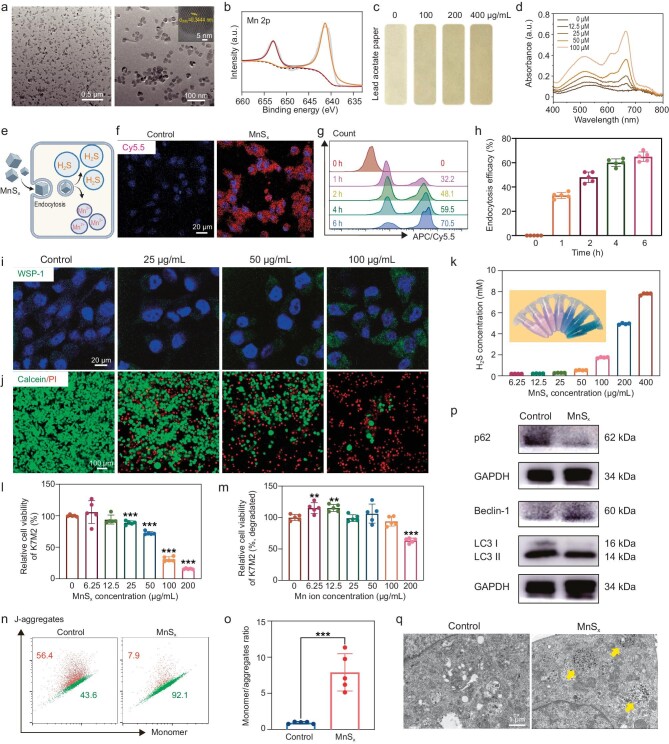
Characterization of MnS_x_ and H_2_S therapy. (a) TEM and high-resolution TEM images of MnS_x_ (scale bars = 0.5 μm, 100 nm and 5 nm). (b) XPS spectrum of MnS_x_. (c) H_2_S release test via a lead acetate method. (d) Ultraviolet-visible-near infrared spectra of the standard curve constructed by NaHS. (e) Schematic diagram of the MnS_x_. Confocal images (f) and flow counting (g) of K7M2 cells incubated with Cy5.5-conjugated MnS_x_ for different time periods (scale bar = 20 μm). (h) Quantitative analysis of cytosolic cells. (i) H_2_S detection of K7M2 after treatment with different concentrations of MnS_x_ (scale bar = 20 μm). (j) Live/dead dual-staining of K7M2 cells after treatment with different concentrations of MnS_x_ (scale bar = 100 μm). (k) Quantification of H_2_S released by different concentrations of MnS_x_. (l, m) MTT assay on K7M2 after treatment with different concentrations of MnS_x_ and Mn. (n, o) Mitochondria membrane potential of K7M2 cells after treatment with MnS_x_. (p) Autophagy-related protein expression in K7M2 cells was detected by western blotting analysis. (q) TEM images of K7M2 (scale bar = 1 μm). The data are presented as the mean ± SD (*n* = 5) in (k), (l) and (m). Statistical significance was calculated by ANOVA with Tukey's *post hoc* test.

H_2_S is a protective gasotransporter that has recently been suggested to be involved in autophagy regulation through specific targets in peroxisulfated cells [32,33]. Thus, we found that H_2_S produced by NaHS enhanced autophagy in K7M2 cells (Fig. S4). However, given that H_2_S is an intracellular gaseous signaling molecule, we synthesized MnS_x_ via a typical high-temperature organic phase approach using MnCl_2_ as a precursor to achieving intracellular enrichment of H_2_S, thereby further enhancing autophagy (Fig. S5). Transmission electron microscopy (TEM) images revealed that MnS_x_ exhibited a cube-like morphology with a well-defined crystal structure (Fig. 2a). Elemental mapping and energy dispersive spectroscopy (EDS) of MnS_x_ confirmed the presence of Mn and S in these nanocubes (Figs S6 and S7). An X-ray powder diffraction (XRD) spectrum revealed a crystal structure corresponding to MnS (Fig. S8). Further analysis to determine the valence states of Mn was conducted using X-ray photoelectron spectroscopy (XPS) (Figs 2b and S9). In the Mn 2p spectra, the binding energies at 641.58 eV and 652.98 eV were assigned to Mn (II) 2p3/2 and Mn (II) 2p1/2, respectively. However, bare MnS_x_ tended to aggregate in aqueous solution, limiting its potential for further biological applications. To address this issue, DSPE-PEG_5000_ was used to modify MnS_x_, enhancing its stability and preventing its aggregation. Dynamic light scattering (DLS) revealed a relatively uniform diameter of 30 to 50 nm after PEGylation, indicating that MnS_x_ was suitable for potential biomedical applications (Fig. S10). Considering the abundant sulfur content, MnS_x_ was anticipated to serve as a donor for releasing H_2_S gas. The generation of H_2_S by MnS_x_ was initially confirmed using lead acetate paper, where higher concentrations of MnS_x_ resulted in darker coloration of the test paper (Fig. 2c). The quantification of H_2_S release was further determined using the zinc acetate-methylene blue (MB) method (Fig. S11). With increasing MnS_x_ concentration, the absorption peak of MB at ∼664 nm gradually increased, indicating a substantial release of H_2_S gas from MnS_x_ depending on the concentration (Fig. 2d). As an endogenous gaseous transmitter, H_2_S is involved in various physiological and pathological states, including cancer, suggesting potential applications of MnS_x_ in cancer therapy through an H_2_S-based approach. Additionally, during this process, MnS_x_ degraded into MnO_x_, as indicated by the XRD pattern similar to that of Mn_3_O_4_, with ∼3% of the Mn^2+^ released (Figs S12 and S13). Through the modification of DSPE-PEG_5000_, the zeta potential indicated that MnS_x_ was positively charged, with a value of 1.75 ± 0.46 mV. The positively charged MnS_x_ is more effective at entering the cell via cytotrophy (Fig. 2e).

We then investigated the uptake behavior of the material by cells. Confocal laser scanning microscopy (CLSM) images revealed Cy5.5-labeled MnS_x_ (red), indicating that MnS_x_ exerts its biological effects within cells through endocytosis (Fig. 2f). Notably, the fluorescence intensity continued to incrementally increase with incubation time, as evidenced by flow cytometry, reaching an endocytosis efficiency of ∼70% at 6 h, demonstrating continuous accumulation of MnS_x_ within the cells (Fig. 2g and h). WSP-1, a commercially available H_2_S probe, transforms into a fluorescent form that emits green fluorescence in the presence of H_2_S. We employed WSP-1 to assay the release of H_2_S from cells in the presence of MnS_x_. The intensity of WSP-1 fluorescence in cells proportionally increased with MnS_x_ concentration, further confirming the ability of MnS_x_ to generate dose-dependent endogenous H_2_S within cells (Fig. 2i).

The therapeutic efficacy of H_2_S is closely linked to dosage, with higher concentrations demonstrating better killing ability against tumor cells [34]. The live and dead cells were co-labeled with calcein acetoxymethyl (Calcein AM) and propidium iodide (PI), respectively (Fig. 2j). The intensity of the red signal progressively enhanced with increasing MnS_x_ concentration, indicating enhanced cell death. The concentration of H_2_S increases gradually with MnS_x_, implying that excess H_2_S has a negative effect on cell proliferation (Fig. 2k). Methyl thiazolyl tetrazolium (MTT) analysis revealed that more than half of the cells died after MnS_x_ treatment. The proliferation of K7M2 cells decreased further with increasing concentrations, with nearly complete cell death observed at 200 μg/mL (Fig. 2l). Comparatively, the killing efficacy of Mn^2+^ at the same concentration was inferior to that of MnS_x_, indicating the enhanced killing efficacy of MnS_x_ in tumor cells (Figs 2m and S14). Further verification of the biocompatibility of MnS_x_ was conducted through hemolysis tests, which indicated that MnS_x_ at 50 μg/mL caused minimal hemolysis while increasing concentrations resulted in erythrocyte rupture (Fig. S15). Subsequent detection of changes in the mitochondrial membrane potential (MMP) in K7M2 cells after MnS_x_ treatment revealed MMP abnormalities induced by MnS_x_, with a significant increase in monomer JC-1, further demonstrating the autophagy-promoting effect of MnS_x_ (Fig. 2n and o).

To examine the effect of MnS_x_ on autophagy, we assessed the expression of Beclin-1, P62 and LC3II/I in K7M2 cells. Beclin-1, recognized as the first mammalian autophagy machinery protein, serves as a molecular scaffold for the assembly of the class III phosphatidylinositol 3 kinase (PI3KC3) complex, controlling autophagy induction and other cellular translocation events [35]. MnS_x_ decreased P62 expression and increased Beclin-1 and LC3II expression, which were associated with autophagy activation compared to those in the control group (Fig. 2p). Furthermore, to elucidate the effect of MnS_x_ on the cellular microstructure, we observed mitochondrial morphology by TEM. Notable morphological changes in K7M2 cells after MnS_x_ treatment included the formation of autophagic vacuoles encapsulating the cytoplasm, mitochondria and endoplasmic reticulum. Double-membraned, giant autophagosomes filled with degraded organelles and autolysosomes were also observed (Fig. 2q, yellow arrows). The number of autophagosomes in K7M2 cells was significantly greater in the MnS_x_-treated group than in the control group (Fig. S16). The MnS_x_ nanoplatforms were successfully designed and fabricated, and the corresponding material characterization of MnS_x_ was completed. It was observed that MnS_x_ was able to enter the cell interior smoothly, resulting in a time-dependent accumulation of intracellular H_2_S. Concurrently, MnS_x_ has been observed to enhance the expression of autophagy-related proteins and elevate the mitochondrial membrane potential, thereby eliciting a favorable cell-killing effect.

### USP8-based autophagy induction in OS by MnS_x_

**Figure 3.**
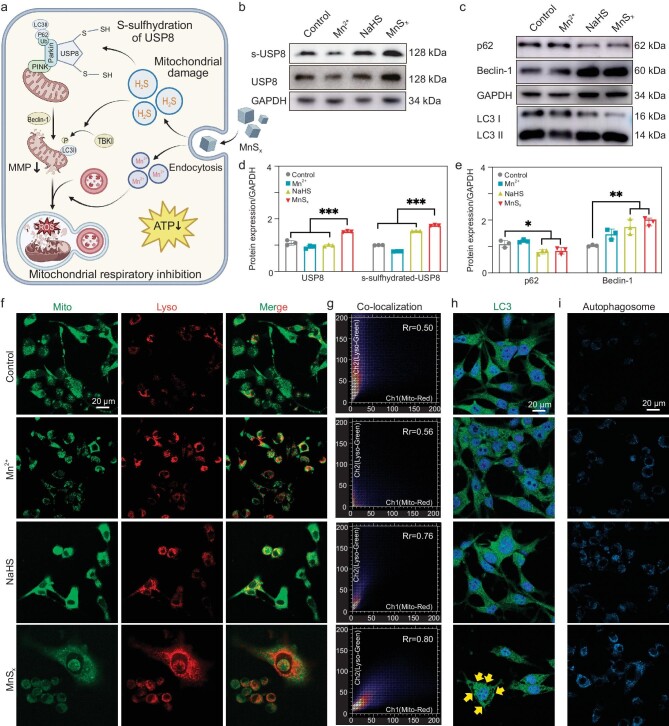
Intracellular H_2_S promotes autophagy in K7M2 mitochondria. (a) MnS_x_ mediates the role of intracellular H_2_S in regulating osteosarcoma mitochondrial autophagy via S-sulfhydration of USP8. (b, d) The expression and quantity of USP8 and S-sulfhydration-USP8 in K7M2 cells were detected by western blotting analysis. (c, e) The expression and quantity of autophagy-related proteins in K7M2 cells were detected by western blotting analysis. (f) Mitochondrial autophagy was detected by CLSM, where green fluorescence represents mitochondria and red fluorescence represents lysosomal fusion. (g) Co-localization of mitochondria with lysosomes. (h) Expression of LC3 in K7M2 cells was detected by CLSM. (i) Detection of autophagosomes (blue) in K7M2 cells by the MDC test. The data are presented as mean ± SD (*n* = 5). Statistical significance was calculated by ANOVA with Tukey's *post hoc* test, scale bar = 20 μm.

Autophagy plays a pivotal role in maintaining mitochondrial homeostasis, ensuring normal cellular function through the timely removal of damaged or dysfunctional mitochondria [36]. Recent studies have highlighted the ability of H_2_S to post-translationally modify proteins by forming persulfur (−SSH) bonds with reactive cysteine residues, a process known as S-sulfhydration [37,38]. Following S-sulfhydration, proteins undergo functional alterations, becoming significant effectors or regulators [39]. Moreover, previous research has identified ubiquitin-specific peptidase 8 (USP8) as a deubiquitinating enzyme that selectively removes atypical K6-linked ubiquitin chains on Parkin, a crucial step for effective Parkin recruitment to depolarized mitochondria [40,41]. Thus, we hypothesized that MnS_x_ likely modulated mitophagy and promoted mitochondrial autophagy by regulating exogenous H_2_S levels and influencing USP8 function through S-sulfhydration (Fig. 3a). Exogenous H_2_S induced S-sulfhydration and upregulated USP8 protein expression in the presence of MnS_x_ (Fig. 3b and d). Furthermore, Western blotting analysis confirmed alterations in the expression of autophagy-related proteins following MnS_x_ treatment, demonstrating a significant increase in cellular autophagy levels (Fig. 3c and e, and Fig. S17). Subsequently, the intracellular mitochondrial and lysosomal conditions were evaluated, which revealed enhanced mitophagosome formation (green fluorescence) and fusion of damaged mitophagosomes with lysosomes (red fluorescence) in the MnS_x_-treated group compared to the control group, as evidenced by mitophagosome and lysosome dyes, respectively (Fig. 3f). This finding supported the notion that MnS_x_ promotes mitochondrial and lysosomal binding (Figs 3g and S18). Previous studies have indicated that LC3II translocates to mitochondria, facilitating the removal of damaged mitochondria via lysosomes [42]. Immunofluorescence against LC3 revealed increased LC3II expression in the mitochondria of MnS_x_-treated K7M2 cells (Figs 3h and S19, yellow arrows), further confirming the activation of cellular autophagy. Subsequent assessment of autophagy using monodansylcadaverine (MDC) staining, which labeled acidic endosomes, lysosomes and autophagosomes, revealed an increase in autophagic vesicles in the NaHS and MnS_x_ treatment groups compared to the control groups (Figs 3i and S20). It could be found that the autophagy-promoting effect of the MnS_x_ nanoplatforms was inhibited by Chloroquine (CQ) (Fig. S21). These findings suggested that MnS_x_ promoted mitochondrial and lysosomal binding and induced mitochondrial autophagy through exogenous H_2_S-mediated activation of USP8 S-sulfhydration.

### cGAS-STING-based activation for OS cellular immunotherapy by MnS_x_

**Figure 4.**
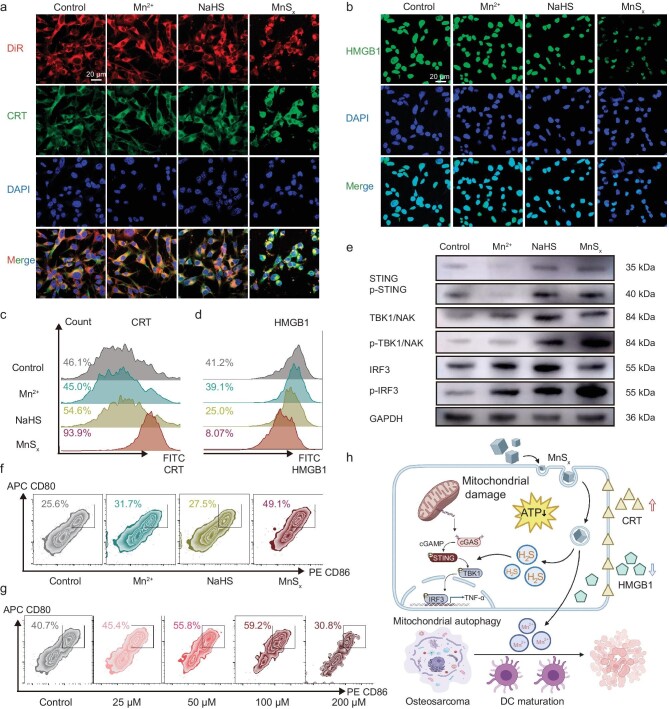
Biological activity of manganese and H_2_S. Fluorescence staining of (a) CRT and (b) HMGB1 in K7M2 cells after various treatments. The expression of (c) CRT and (d) HMGB1 in K7M2 cells was detected by flow cytometry. (e) The expression of STING-related proteins in HeLa cells was detected by western blotting analysis. (f) DC maturation after various treatments. (g) DC maturation after treatment with different concentrations of MnS_x_. (h) MnSx mediates the intracellular cGAS-STING signaling axis to regulate the immune environment of OS. The data are presented as mean ± SD (*n* = 5). Statistical significance was calculated by ANOVA with Tukey's *post hoc* test, scale bar = 20 μm.

**Figure 5.**
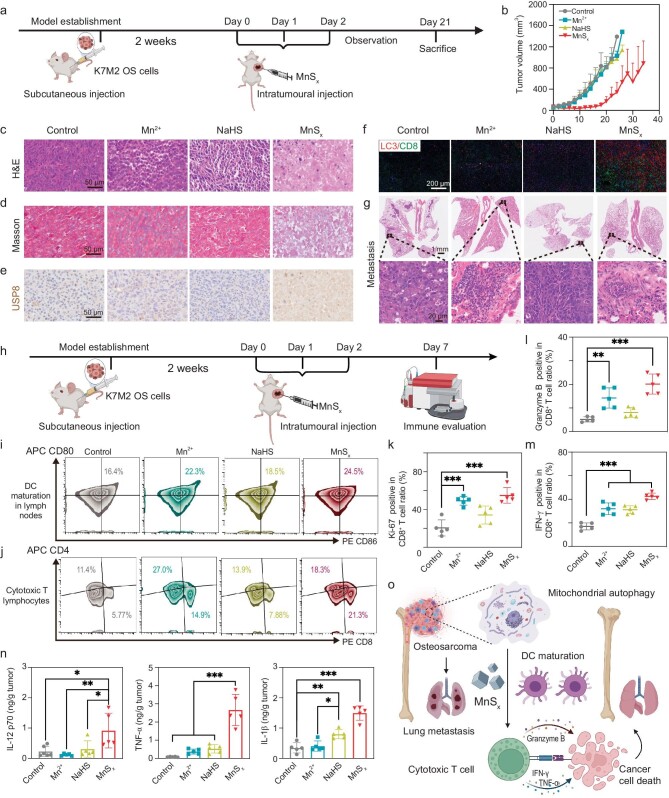
*In vivo* antitumor efficacy and immune evaluation of MnS_x_. (a) Schematic illustration of the *in vivo* treatment procedure for Balb/c mice. (b) Average tumor growth curves of the mice in the treatment groups. Images of tumor slices after (c) H&E staining and (d) Masson staining (scale bar = 50 μm). (e) Images of tumor sections after immunohistochemistry for USP8 (scale bars = 50 μm). (f) Images of tumor slices after immunofluorescence staining of CD8^+^ and LC3^+^ cells (scale bar = 200 μm). (g) Lung metastasis of mice in the treated groups (scale bars = 1 mm and 20 μm). (h) Schematic illustration of the *in vivo* immune response. (i) Representative flow cytometric analysis of DC maturation (CD80^+^ CD86^+^) in lymph nodes, gating on CD11c^+^ cells. (j) CD8^+^ T cells among CD3^+^ CD45^+^ T cells in the tumors. (k) Ki-67^+^ CD8^+^ T cells among CD3^+^ CD45^+^ T cells in tumors. (l) Granzyme^+^ CD8^+^ T cells among CD3^+^ CD45^+^ T cells in tumors. (m) IFN-γ^+^ CD8^+^ T cells among CD3^+^ CD45^+^ T cells in tumors. (n) Detection of the cytokines IL-12 p70, IL-1β and TNF-α in tumors. (o) MnSx-mediated mitochondrial autophagy regulates the tumor immune microenvironment to kill OS. The data are presented as mean ± SD (*n* = 5). Statistical significance was calculated by ANOVA with Tukey's *post hoc* test.

The investigation delved into the expression of the ‘eat-me’ signal calreticulin (CRT), which swiftly translocates from the endoplasmic reticulum to the cell surface within hours of immunogenic cell death (ICD) initiation, serving as a recognizable cue for phagocytes [43,44]. Both CLSM images and flow cytometry analyses revealed MnS_x_-induced CRT translocation (Fig. 4a and c, and Fig. S22). Afterwards, the release of ‘find me’ signals, such as adenosine triphosphate (ATP) and high mobility group box 1 (HMGB1), was also assessed [45]. HMGB1 binds to Toll-like receptor 4 on immature DCs membranes, fostering DC maturation and antigen presentation to activate CTLs. Moreover, ATP recruits DCs, which in turn activate NLRP3 inflammatory vesicles for further elimination of cancer cells [46,47]. As expected, MnS_x_ treatment amplified ‘find me’ signaling, ATP and HMGB1 release, inducing autophagy in K7M2 OS cells (Fig. 4b and d, and Fig. S23). Additionally, ATP was released in an autophagy-dependent manner, resulting in a significant decrease in intracellular ATP, consistent with the robust autophagy levels observed after prior MnS_x_ treatment (Fig. S24). Subsequent exploration investigated the influence of MnS_x_ on the maturation of bone-marrow-derived dendritic cells (BMDCs). Compared to those in the control group, the supernatants of cells treated with MnS_x_ displayed elevated expression of CD80 and CD86 in BMDCs, indicating enhanced maturation levels of BMDCs (Figs 4f and S25). Moreover, a progressive increase in the maturation level of BMDCs was found in line with increasing MnS_x_ concentration (Figs 5g and S26). According to Matzinger's danger signaling theory, fragments generated from excessive autophagy activate antigen-presenting cells (APCs), triggering an immune response [48]. Thus, we hypothesized that excessive cellular autophagy after MnS_x_ treatment played a pivotal role in OS treatment.

Furthermore, the effect of MnS_x_ on the cellular cGAS-STING signaling axis, which detects pathogen deoxyribonucleic acid (DNA) and stimulates an innate immune response, was investigated [49]. cGAS and STING have been shown to mediate autophagy induction and lysosome-dependent cell death independently of interferons [50,51]. Our results validated the ability of MnS_x_ to promote STING phosphorylation, facilitating downstream phosphorylation of TBK1 and IRF3, which further activated the STING pathway, promoting intracellular mitochondrial autophagy (Figs 4e and S27). Above all, MnSx triggers CRT translocation and the release of ATP and HMGB1, further propelling the cGAS-STING signaling axis, which in turn activates mitochondrial autophagy to target and eliminate tumor cells. ATP induces DC maturation and antigen presentation, activating CTLs for further tumor destruction.

### *In vivo* antitumor efficacy of MnS_x_

Following the promising *in vitro* antitumor response to K7M2, we further validated the *in vivo* efficacy of MnS_x_ against tumors (Fig. 5a). Compared to the control group, both the Mn^2+^ and NaHS groups exhibited a certain degree of tumor growth inhibition, possibly attributed to H_2_S release from NaHS and the tumor-inhibitory effect of manganate. Notably, tumors were significantly suppressed following MnS_x_ treatment across all four treatment groups (Figs 5b and S28). Histological analysis through hematoxylin-eosin (H&E) staining revealed pronounced tumor necrosis with crumpled nuclei post-MnS_x_ treatment (Fig. 5c), along with localized collagen hyperplasia observed via Masson staining (Fig. 5d). Immunohistochemical staining for USP8 revealed a marked increase in USP8 expression in tumors post-MnS_x_ treatment, consistent with the *in vitro* results (Figs 5e and S29). Immunofluorescence staining further elucidated the role of autophagic flow activation in MnS_x_-mediated tumor antagonism, with increased fluorescence of LC3 and CD8 detected after MnS_x_ treatment (Figs 5f and S30). Clinically, OS is highly aggressive and typically associated with lung metastasis. K7M2 OS cells, which are derived from secondary lung metastases of the K7 OS cell line, are known for their high invasiveness, with a lung metastasis rate exceeding 90% in mice [52]. Hence, we evaluated lung metastases in mice through lung tissue sectioning and H&E staining. Despite the superior tumor inhibitory effect of MnS_x_, it did not completely prevent lung metastasis in K7M2-homozygous mice, with metastatic nodules still evident in lung tissue sections after MnS_x_ treatment (Fig. 5g). Analysis of the number of lung nodules revealed that MnS_x_ treatment significantly inhibited metastasis compared to that in the other groups, possibly due to autophagy induction within the tumor following the treatment (Fig. S31). However, the observed recurrence and metastasis in the Mn^2+^ and NaHS alone treatment groups were not satisfactory, highlighting the need for further strategies for OS eradication. H&E staining of major organs after MnS_x_ treatment at different time points revealed no significant histological damage, indicating the biosafety of MnS_x_ as a highly effective anti-tumor nanoplatform (Fig. S32). Additionally, blood collected for biochemical testing on days 1 and 30 post-dose showed all blood indices within the normal range, with liver (alanine aminotransferase, glutamine aminotransferase, alkaline phosphatase, total bilirubin) and kidney (creatinine, urea nitrogen) functional indices remaining within reference ranges (Fig. S33). Consequently, MnS_x_ exhibits a favorable tumor-killing effect while simultaneously demonstrating minimal toxicity to vital organs.

### *In vivo* immune assessment and tumor-microenvironment modulation by MnS_x_

Due to the promising therapeutic efficacy of MnS_x_ on OS *in vivo*, we evaluated the immune response following MnS_x_ treatment (Fig. 5h). On day 7, the mice were euthanized, and the tumors and lymph nodes were collected to prepare single-cell suspensions for immune cell analysis via flow cytometry (Fig. S34). DCs are crucial APCs, and their maturation in proximal lymph nodes significantly increased after intra-tumoral MnS_x_ injection, indicating robust antigen presentation and immune system activation *in vivo* (Figs 5i and S35). In mechanistic terms, Mn^2+^ enhances DC and macrophage maturation, as well as tumor-specific antigen presentation, strengthens cytotoxic T lymphocyte differentiation, activation and NK cell activity, and increases memory CTLs [20]. T lymphocytes are key components of OS cellular immunity. Different T cell subtypes exert diverse functions in tumors, with studies highlighting a strong correlation between CTLs and improved prognosis in cancer patients [53]. Enhanced CTL infiltration into the OS tumor microenvironment (TME) has been linked to enhanced efficacy of cancer immunotherapy [54]. Notably, MnS_x_ treatment significantly promoted CTL infiltration in OS, with an average infiltration rate of ∼18%, compared to ∼5% in the control group (Figs 5j and S36).

Further evaluation of CTL revealed that MnS_x_ did not impede CTLs proliferation; instead, it increased the percentage of Ki-67-positive CTLs by up to 50% (Figs 5k and S37). Granzyme B, the most abundant serine protease in CTLs, plays a crucial role in eliminating harmful target cells, including tumor cells [55]. Our results demonstrated a positive effect of MnS_x_ treatment on enhancing granzyme B expression in CTLs (Figs 5l and S38). Moreover, MnS_x_-induced inflammation led to high expression of interferon-gamma (IFN-γ) in CTLs and upregulated antigen presentation in DCs (Figs 5m and S39). IFN-γ signaling is a key immunogenic pathway that plays a decisive role in spontaneous and ICB-induced anti-tumor immunity. Host IFN-γ signaling supports tumor antigen presentation, activation of APCs and recruitment of effector T-cells, and has a direct impact on tumor cell proliferation and survival [56]. Immunological assessments indicate that OS may exhibit a deficiency in mature antigen-presenting cells. It may therefore be beneficial to promote the maturation of antigen-presenting cells by MnS_x_-induced autophagy in order to trigger a high level of immunogenic death of OS cells, thereby recruiting immune cells to play a functional role.

Subsequent verification of inflammatory factor expression in tumor supernatants by enzyme linked immunosorbent assay (ELISA) corroborated the immune evaluation, showing relatively down-regulated levels of anti-inflammatory factors such as IL-10 (Fig. S40). Effector cytokines such as IFN-γ and the inflammatory mediators IL-1β and TNF-α were secreted in large quantities, thereby promoting further DC maturation (Figs 5n and S41). Moreover, IL-12 p70 produced by DCs as well as other APCs can promote the proliferation of T-cells and NK-cells on the one hand, and their production of IFN-γ on the other hand, further promoting the formation of CTLs. In general, as demonstrated *in vitro*, the intratumoral administration of MnS_x_ results in a considerable degree of cellular autophagy within the tumor, facilitates the liberation of dampness, and subsequently triggers the activation of the APCs, which in turn generate a substantial number of effector cytokines. This ultimately results in the antigen presentation and maturation of DCs within the lymph nodes, thereby activating the *in vivo* immune system and increasing the infiltration of intratumoral CTLs, which can exert a killing effect on the OS (Fig. 5o).

### USP8-based autophagy induction of MnS_x_ to cure OS

**Figure 6.**
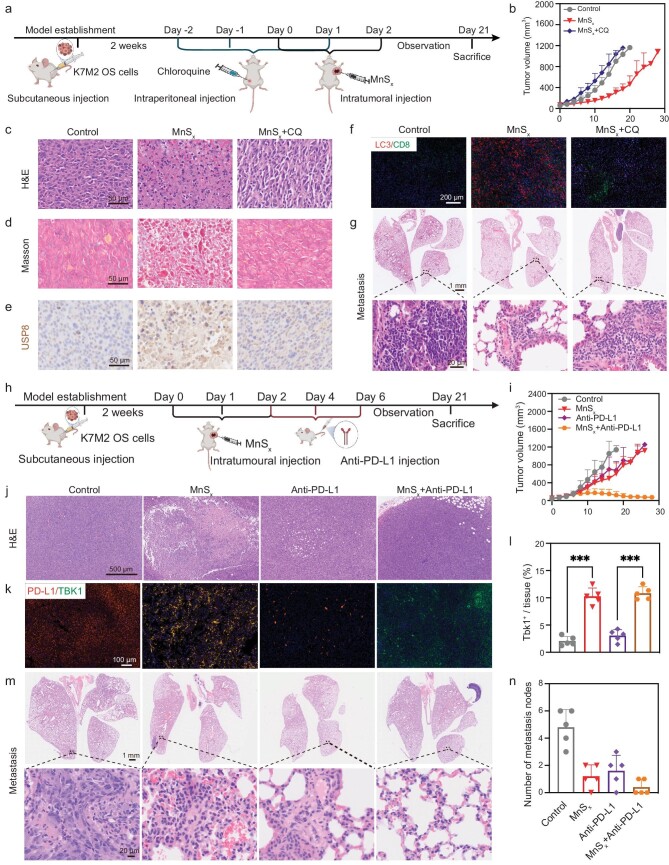
*In vivo* autophagy blockade and α-PD-L1 combination therapy. (a) Schematic illustration of the *in vivo* treatment procedure in combination with autophagy blockade in Balb/c mice. (b) Survival curves of the mice after the treatments. Images of tumor slices after (c) H&E staining and (d) Masson staining (scale bar = 50 μm). (e) Images of tumor sections after immunohistochemistry for USP8 (scale bar = 50 μm). (f) Images of tumor slices after immunofluorescence staining of CD8^+^ and LC3^+^ cells (scale bar = 200 μm). (g) Lung metastasis of mice for the treated groups (scale bars = 1 mm and 20 μm). (h) Schematic illustration of the *in vivo* treatment procedure involving combination treatment with α-PD-L1 in mice. (i) Average tumor growth curves of the mice in the treatment groups. (j) Images of tumor slices after H&E staining (scale bar = 500 μm). (k) Images of tumor slices after immunofluorescence staining of TBK1^+^ and PD-L1^+^ cells (scale bar = 100 μm). (l) Quantitative analysis of the percentage area of immunofluorescence staining for TBK1. (m) Lung metastasis of mice for the treated groups (scale bars = 1 mm and 20 μm). (n) Quantitative analysis of lung metastases. The data are presented as mean ± SD (*n* = 5). Statistical significance was calculated by ANOVA with Tukey's *post hoc* test.

Autophagy was found to be upregulated in *in vitro* experiments following MnS_x_ treatment, indicating the potential of this therapy to induce tumor cell death by activating excessive autophagy. Chloroquine, a commonly prescribed autophagy inhibitor, can disrupt lysosomal function and suppress the fusion of lysosomes and autophagosomes, thereby effectively inhibiting autophagic flux [57]. Thus, CQ was used to inhibit autophagy to further elucidate the effect of MnSx treatment on autophagic flux in OS (Fig. 6a). The results revealed that tumors were not inhibited in the control group, while tumor growth was significantly restrained in the MnS_x_ group. However, the anti-tumor effect of the combination of CQ and MnS_x_ was notably diminished (Fig. S42). The tumor growth curves of Balb/c mice were illustrated following different treatments (Figs 6b and S43). H&E staining demonstrated that CQ counteracted the inhibitory and cytotoxic effects of MnS_x_ on tumors (Fig. 6c), and Masson staining corroborated these findings (Fig. 6d). Furthermore, immunohistochemical staining for USP8 revealed a significant increase in USP8 expression in tumors after MnS_x_ treatment, which was attenuated by CQ (Figs 6e and S44). Surprisingly, the fluorescence of LC3 following MnS_x_ treatment was similarly suppressed by CQ (Figs 6f and S45). Moreover, MnS_x_ treatment significantly reduced the rate of lung metastasis of the tumor, as observed through sectioning and H&E staining of the lung tissues. However, the rate of lung metastasis was significantly elevated in the CQ group (Figs 6g and S46). Overall, these findings suggest that MnS_x_-induced autophagy plays a crucial role in inhibiting tumor growth and metastasis in OS and that inhibition of autophagy by CQ attenuates the therapeutic efficacy of MnS_x_, highlighting the importance of autophagic flux modulation in MnS_x_-based therapy.

### Combination with ICB therapy for OS

However, the usual inability of T cells to eradicate tumors in the native TME may lie in the fact that anti-tumor T cells gradually enter a dysfunctional state during cancer progression, making tumor immune evasion possible [13,58]. Evidence suggests that α-PD-L1 is a signaling molecule expressed on the surface of tumor cells and that α-PD-L1-based ICBs often results in treatment failure due to the inherent resistance of tumor cells [59]. For this reason, we subsequently explored the anti-tumor effects of MnS_x_ in combination with α-PD-L1 (Fig. 6h). Tumors in the control group were not significantly inhibited. Unsurprisingly, tumor growth was significantly inhibited in the MnS_x_ and α-PD-L1 groups, while α-PD-L1 combined with MnS_x_ had the greatest anti-tumor effect (Figs 6i and S47). H&E staining revealed that the nuclei inside the tumor had shrunk, suggesting that the combination therapy was more damaging to the tumor cells than either MnS_x_ alone or α-PD-L1 alone (Fig. 6j). MnS_x_ treatment suppressed α-PD-L1 fluorescence and enhanced TBK1 fluorescence, suggesting that MnS_x_ can promote the activation of TBK1 downstream of the Sting-related pathway, while the combination treatment did not attenuate this activation (Fig. 6k and l). Moreover, the combination treatment further suppressed the expression of α-PD-L1 within the tumor, suggesting that more tumor cells died, and that cell proliferation was significantly inhibited by the α-PD-L1 combination treatment (Fig. S48). Similarly, treatment with MnS_x_ combined with α-PD-L1 significantly reduced the rate of lung metastasis of the tumor, as determined by whole lung tissue sectioning (Fig. 6m and n). The results demonstrated that the combination of MnS_x_ with an α-PD-L1 immune checkpoint inhibitor resulted in complete remission of OS and the eradication of lung metastatic lesions.

## CONCLUSIONS

In conclusion, our findings provide compelling evidence that MnS_x_ is a dual functional metal nanoplatform for the treatment of OS through multiple mechanisms. Bioinformatics analyses of clinical OS samples revealed impaired autophagy, while the inhibition of mitochondrial autophagy in OS was further verified at the transcriptomic level. MnS_x_ effectively eradicates OS by generating H_2_S intracellularly via endocytosis. This lethal effect of MnS_x_ can be attributed, in part, to the activation of USP8 by intracellular H_2_S via S-sulfation, which facilitated the fusion of mitochondria and lysosomes and triggered mitochondrial autophagy. Additionally, MnS_x_ activated the cGAS/STING pathway, which amplified the cellular autophagic response, further accelerating the tumor-killing effect. Moreover, MnS_x_ activated intratumoral autophagy via USP8, and the released Mn^2+^ also promoted the maturation of DCs, leading to the activation of cytotoxic T lymphocytes and subsequent elimination of OS cells. The autophagy inhibitor CQ suppressed the tumor killing effect, and the combination therapy with α-PD-L1 immune checkpoint inhibitors achieved complete remission of OS. These mechanistic insights shed new light on the therapeutic potential of MnS_x_ in OS treatment. This study lays the groundwork for exploring the clinical application of dual-functional nanoplatforms in OS immunotherapy and demonstrates that using nanoplatforms to enhance the biological effects of tumors while modulating the immune microenvironment is a promising strategy. These insights offer promising avenues for the development of multifaceted therapeutic platforms tailored for the treatment of OS, addressing critical challenges in current treatment paradigms.

## ETHICAL STATEMENTS

The First Affiliated Hospital of Soochow University has approved the animal experiments.

## Supplementary Material

nwaf002_Supplemental_File
