# Supplementary material for: Dual functional nanoplatforms potentiate osteosarcoma immunotherapy via microenvironment modulation
Source: Natl Sci Rev. 2025 Jan 10;12(3):nwaf002. doi: 10.1093/nsr/nwaf002 (PMC11812574; doi:10.1093/nsr/nwaf002)
Supplement: nwaf002_Supplemental_File [file nwaf002_supplemental_file.pdf]

Supporting Information

Dual Functional Nanoplatforms Potentiate Osteosarcoma Immunotherapy via Microenvironment Modulation

Shunyi Lu[1,2#], Yuqi Yang[2#], Zhuorun Song[1,2#], Jie Cao[2], Zhihui Han[2], Linfu Chen[2], Yunfei He[3], Jiayi Wang[4], Yun Teng[1], Zengli Zhang[5], Jun Zou[1*], Jun Ge[1,2*], Huilin Yang[1*] and Liang Cheng[2*]

[1]Department of Orthopedic Surgery, The First Affiliated Hospital of Soochow University, Suzhou, 215123, China

[2]Institute of Functional Nano & Soft Materials (FUNSOM), Jiangsu Key Laboratory for Carbon-Based Functional Materials & Devices, Soochow University, Suzhou, 215123, China

[3]The First Affiliated Hospital of Soochow University, Suzhou, 215123, China

[4]Shanghai Jiao Tong University Affiliated Sixth People's Hospital, Shanghai 200233, China

[5]Department of Environmental Health School of Public Health, Soochow University, Suzhou 215123, China

[#]These authors contribute equally to this work.

*Corresponding authors: jzou@suda.edu.cn; gejun115@suda.edu.cn; hlyang@suda.edu.cn; lcheng2@suda.edu.cn

1. Experiment and Methods

Synthesis of MnSx nanoparticles.

Taking $MnCl_2$ as a precursor, MnS_x NPs were fabricated via a kind of high-temperature organic-phase approach. Specifically, 250 mg of $MnCl_2$ (2 mmol) was dispersed in 20 mL of OM and 15 mL of ODE in a three-necked flask under magnetic stirring. This mixture was heated to 140 ℃ and stirred for 20 mins under a N_2 atmosphere to remove the water and oxygen. Then, this mixture was heated to 260 ℃ at this temperature for 20 mins. Next, a S/OM solution, prepared by dissolving 128 mg of S powder (4 mmol) in 5 mL of OM, was injected into the flask at 260 ℃ for 20 mins. After being cooled down to room temperature, the MnS_x NPs were washed with cyclohexane and ethanol by three times.

To enhance the biocompatibility, DSPE-PEG-NH_2 (MW = 5000, Ponsure, China) was employed to modify MnS_x. Detailly, 50 mg of DSPE-PEG-NH_2 was dissolved in 4 mL of chloroform, followed by the addition of 10 mg of MnS_x dispersed in 1 mL of chloroform under sonication for 10 mins. N_2 was used to dry the solution to obtain the final solid sample, which was centrifugated at 3000 rpm for 5 min, dispersed in deionized water and stored at 4 ℃ with N_2 protection for future use.

Characterization

The morphologies of MnS_x were characterized by transmission electron microscopy (TEM, tecnai F20). The crystal structure and surface chemical composition of MnS_x were measured by X-ray diffraction (XRD, Panalytical Empyrean) and X-ray photoelectron spectroscopy (XPS, ESCALab

250Xi). The absorption spectra were obtained by a UV-vis-NIR spectrophotometer (Thermo50 UV-vis-NIR spectrophotometer, Thermo Scientific). The absolute concentration of Mn ions was measured by inductively coupled plasma-optical emission spectroscopy (ICP-OES, Avio 200).

To observe H_2S generation, a piece of lead acetate test paper was used to cover on the top of the culture dish to test whether H_2S was generated from 0, 50, 100, or 200 μg/mL MnS_x within 120 min at 37 ℃.

For quantitative determination of H_2S generation, MnS_x was mixed with 1 mL of a zinc acetate/sodium acetate mixture (4:1 mass ratio). Then, DMPD ·2HCl and $FeCl_3$ were added to form MB. After a 15 min of incubation at 37 ℃, the absorbance at ~664 nm was detected, and a standard curve of NaHS was established to determine the concentration of H_2S.

Data collection and scRNA-Seq data analysis

Single-cell RNA sequencing (scRNA-seq) of OS tissue from GSE152048 and scRNA-seq of healthy bone tissue from GSE169396 were obtained from the NCBI Gene Expression Omnibus (https://www.ncbi.nlm.nih.gov/geo). For 7 primary OS patients in GSE152048 and 4 normal samples in GSE169396, batch effects between samples were removed using harmony, and retained cells with nFeature_RNA greater than 200 and less than 10,000, nCount_RNA greater than 500 and less than 80,000, and percent.mt less than 20 were retained. A total of 109,674 cells were obtained for subsequent analysis, degradation and clustering were performed with Seurat, and trajectory analysis was conducted with Monocle.

Cellular experiments

The K7M2 murine osteosarcoma cell line was obtained from the American Type Culture Collection (ATCC) and cultured in the standard cell culture medium at the condition of 37 ℃, 5% CO_2.

For cellular uptake of MnS_x, K7M2 cells were incubated with Cy5.5-labeled MnS_x (50 μg/mL) for 12 h. All images were acquired by confocal laser scanning microscopy (CLSM, Zeiss LSM 800). For flow cytometry, the collected cells were first blocked with 5% FBS (JYK-FBS-303, INNER MONGOLIA JIN YUAN KANG BIOTECHNOLOGY CO., LTD) for 30 mins. After washing with PBS three times, the cells were then incubated with secondary antibody (Alexa Fluor 488, Abcam, USA) for 30 min according to the recommended protocol. The collected cells were washed with PBS and incubated with C6 plus (n = 3).

For H_2S detection *in vitro*, K7M2 cells were first incubated with the WSP-1 probe (50 μM) for 30 mins. After removing the probe and washing with PBS, K7M2 cells were incubated with MnS_x (0, 25, 50 and 100 μg/mL) for 1 h. All the images were acquired by CLSM. The sulfide concentration was detected using p-aminodimethylaniline spectrophotometry.

For live/dead dual-staining *in vitro*, K7M2 cells were incubated with MnS_x (0, 25, 50 and 100 μg/mL) for 12 h, then incubated for 4 h again, followed by staining with calcein AM (AM) and

propidium iodide (PI) for 30 min according to the recommended protocol. All the images were acquired by CLSM.

For *in vitro* cell viability, the standard 3-(4,5-dimethyl-2-thiazolyl)-2,5-diphenyl-2-htetrazolium bromide (MTT) assay was performed to determine the relative cell viability after different treatments.

To measure the mitochondrial membrane potential, K7M2 cells were incubated with MnS_x (50 μg/mL) for 12 h, followed by staining with the lipophilic cationic probe 5,5',6,6'-tetrachloro-1,1',3,3'-tetraethyl-imida-carbocyanine iodide (JC-1) for 20 min according to the recommended protocol (n = 3). The collected cells were washed and tested with C6 plus. Other groups included the control group, Mn^{2+} group, and NaHS group.

To verify the relative protein expression associated with autophagy *in vitro*, K7M2 cells were incubated with MnS_x (50 μg/mL) for 12 h and then collected and suspended in 4 ℃ cell lysis buffer for western blotting according to the recommended protocol.

The relative RNA expression of cell autophagy-related genes *in vitro* was verified. The total RNA was extracted, and reverse transcription was performed using a RNeasy Mini Kit (BioTeke, China) and PrimeScript RT kit (TAKARA, USA), respectively, according to the instructions. The sequence-specific primers used are listed in Table 1. The target PCR amplification products were verified by gel purification (Qiagen). RNA was quantified using the Bio-RAD CFX Connect

Real-Time PCR System (Bio-Rad). GAPDH was used as an internal control. The data were analyzed by relative quantification ($2^{-\Delta\Delta Ct}$).

To verify the microstructure of cellular autophagy *in vitro*, K7M2 cells were incubated with MnS_x (50 μg/mL) for 12 h. The cells were then collected and fixed in electron microscopy fixative, and transmission electron microscopy (TEM) was performed according to the recommended protocol.

To verify the relative protein expression associated with autophagy *in vitro,* K7M2 cells and Hela cells were incubated with MnS_x (50 μg/mL) for 12 h and then collected and suspended in 4 ℃ cell lysis buffer for western blotting according to the recommended protocol. Antibodies were as follows: STING antibody (#13647, CST), p-STING antibody (#50907, CST), TBK1 antibody (#38066, CST), p-TBK1 antibody (#5483, CST), IRF3 antibody (#4302, CST), p-IRF3 antibody (#50907, CST), Beclin-1 antibody (#833944, CST), p62 antibody (ab109012, abcam), LC3 antibody (ab192890, abcam), USP8 antibody（P012660, Epizyme). The other groups included the control, Mn^{2+}, and NaHS groups.

To test S-sulfhydration expression, K7M2 cells were incubated with MnS_x (50 μg/mL) for 12 h, homogenized in HEN buffer supplemented with 100 μM deferoxamine, and then centrifuged at 13,000 g for 30 min. The ceell lysate (240 μg) was added to blocking buffer at 50°C for 20 min with frequent vortexing. MMTS (Sigma, USA) was then removed with acetone, and the proteins were precipitated at -20 ℃ for 20 mins. After acetone removal, the proteins were resuspended in HENS buffer. Then, 4 mM biotin-HPDP (Sigma, USA) in DMSO without ascorbic acid was added to the

suspension. After incubation at 25 ℃ for 3 h, the biotinylated proteins were precipitated with streptavidin-agarose beads, which were then washed with HENS buffer. Biotinylated proteins were eluted by SDS-polyacrylamide gel electrophoresis (SDS-PAGE, Sigma, USA) sample buffer and then analyzed by Western blotting. The other groups included the control, Mn^{2+}, and NaHS groups.

To analyze mitochondrial autophagy, K7M2 cells were inoculated in 35 mm dishes with MnS_x (50 μg/mL) for 12 h, followed by incubation with MitoTracker (200 nM, Beyotime, China) and Lyso Tracker (50 nM, Beyotime, China) for 30 min separately at 37 ℃ in an incubator. Then, the cells were washed with PBS and observed using CLSM. The co-localization of mito- and lyso-fluorescence in the acquired images was analyzed using ImageJ (NIH Bethesda, MD). The other groups included the control, Mn^{2+}, and NaHS groups.

To test LC3 expression, K7M2 cells were incubated with MnS_x (50 μg/mL) for 12 h. The collected cells were first blocked with 5% FBS and then incubated with primary antibody (LC3, Abcam, USA) for 3 h. After washing with PBS three times, the cells were then incubated with secondary antibody (Alexa Fluor 488, Abcam, USA) for 1 h according to the recommended protocol. All the images were acquired by CLSM. The other groups included the control, Mn^{2+}, and NaHS groups.

For observation of autophagic vacuoles by the MDC assay, K7M2 cells were incubated with MnS_x (50 μg/mL) for 12 h. Then, the cells were incubated with MDC (50 μM) in PBS at 37°C for 30 mins. Autophagic vacuoles were analyzed using CLSM. The other groups included the control, Mn^{2+}, and NaHS groups.

To test CRT expression, the K7M2 cells were incubated with MnS_x (50 μg/mL) for 12 h. For flow cytometry, the collected cells were first blocked with 5% FBS and incubated with a primary antibody (CRT, Abcam, USA) for 30 mins. After washing with PBS three times, the cells were then incubated with secondary antibody (Alexa Fluor 488, Abcam, USA) for 30 min according to the recommended protocol. The collected cells were washed with PBS and tested by C6 plus (n = 3). For CLSM, the collected cells were first incubated with DiR (Beyotime, China) and then blocked with 5% FBS. The cells were incubated with primary antibody (CRT, Abcam, USA) for 3 h. After washing with PBS three times, the cells were then incubated with secondary antibody (Alexa Fluor 488, Abcam, USA) for 1 h according to the recommended protocol. All the images were acquired by CLSM. The other groups included the control group, Mn^{2+} group, and NaHS group.

To test HMGB1 expression, K7M2 cells were incubated with MnS_x (50 μg/mL) for 12 h. For flow cytometry, the collected cells were first blocked with 5% FBS and incubated with primary antibody (HMGB1, Abcam, USA) for 30 mins. After washing with PBS three times, the cells were then incubated with secondary antibody (Alexa Fluor 488, Abcam, USA) for 30 min according to the recommended protocol. The collected cells were washed with PBS and tested by C6 plus (n = 3). For CLSM, the collected cells were first blocked with 5% FBS, and incubated with 1% Triton/PBS solution and then primary antibody (HMGB1, Abcam, USA) for 3 h. After washing with PBS three times, the cells were then incubated with secondary antibody (Alexa Fluor 488, Abcam, USA) for 1 h according to the recommended protocol. All the images were acquired by CLSM. The other groups containing included the control, Mn^{2+}, and NaHS groups.

To measure ATP release, the K7M2 cells were incubated with MnS_x (50 μg/mL) for 12 h (n = 5). The cells were lysed under ultrasound (with a power of 25%, sonication for 3 S, and pause for 10 S; this process was repeated 30 times) on ice and centrifuged to collect the supernatant. The collected supernatants were measured with an ATP Assay Kit (Beyotime, China) according to the manufacturer's protocols. The other groups included the control, Mn^{2+}, and NaHS groups.

To promote DCs maturation, bone marrow-derived dendritic cells (BMDCs) were collected from the bone marrow of female mice (C57BL/6, 6-8 weeks old) and cultured with granulocyte macrophage-colony stimulating factor (GM-CSF, Peprotech, USA) according to the recommended protocol. The supernatants of MnS were collected and quantified by ICP. Then, BMDCs were incubated with lipopolysaccharide (LPS, 50 ng/mL, Beyotime, China) and different concentrations of Mn^{2+} (0, 25, 50, 100 and 200 μM) for 12 h (n = 3). The maturation of BMDCs was determined by C6 plus, and matured DCs were $CD11c^+$, $CD80^+$ and $CD86^+$. All the antibodies purchased from Biolegend used in our experiments were diluted 200 times. The other groups included the control, Mn^{2+}, and NaHS groups.

In vivo study

The animal experiments in this study were conducted under the protocols approved by the Soochow University Laboratory Animal Center. For whole transcriptome sequencing experiments, K7M2 tumor ($4*10^6$) were injected unilaterally alongside the tibia in female Balb/c mice, and when the

tumor volume reached ~1000 mm^3, the Balb/c mice were sacrificed after X-ray photography, and the tumor tissues and contralateral healthy muscle tissues were snap-frozen in liquid nitrogen for whole transcriptome gene sequencing. Total RNA was harvested from the BMSCs with TRIzol reagent. Differential expression analysis was conducted with the DESeq R package (2012).

Female Balb/c mice bearing subcutaneous K7M2 tumors ($5*10^6$) were chosen as the experimental tumor model. When the tumor volume reached ~100 mm^3, the Balb/c mice were randomized into four groups (n = 5 per group) with the following treatments: (1) control, PBS, (2) intratumoral injection of $MnCl_2$, (3) intratumoral injection of NaHS, and (4) intratumoral injection of 5 mg/kg MnS_x. Injections were carried out on day 0 and day 3. Injections were carried out on day 0, day 1 and day 2. The tumor volume and body weight were recorded every 2 days. Tumor volume was calculated based on the following formula: $width^2 \times length/2$. After the treatment course, the mice were sacrificed at random. The tumors were removed for histopathological analysis including H&E staining and Masson staining. H&E staining of bilateral lungs was utilized to visualize tumor lung metastases. Immunohistochemical staining (for USP8) of the tumors was subsequently performed. Immunofluorescence staining ($LC3^+$ and $CD8^+$ cells) was also detected after treatment.

Immune Evaluation

When the tumor volume reached ~100 mm^3, the Balb/c mice were randomly divided into four groups (n = 5 per group) with the following treatments: (1) control, PBS, (2) intratumoral injection of $MnCl_2$, (3) intratumoral injection of NaHS, and (4) intratumoral injection of 5 mg/kg MnS_x.

Injections were carried out on day 0, day 1 and day 2. The mice were sacrificed on day 7. The tumor tissue and the lymph node near the tumor were processed into single-cell suspensions for flow cytometry detection of a variety of immune cells. In addition, the tumor supernatants were collected, and the levels of various cytokines, such as IL-1β, IFN-γ, TNF-α, IL-12 p70 and IL-10, were analyzed (TragetMol, USA).

Autophagy blockade therapy

After the tumors grew to ~100 mm^3, the Balb/c mice were randomized into three groups (n = 5 per group) with the following treatments: (1) control, PBS, (2) intratumoral injection of 5.0 mg/kg of MnS_x, and (3) CQ+MnS_x, intraperitoneal injection of 30 mg/ml of chloroquine (CQ) and intratumoral injection of 5.0 mg/kg MnS_x. MnS_x was injected on days 0, 1 and 2, while CQ (TargetMol, T8689, China) was injected on days -2, -1, and 1. Tumor volume and body weight were recorded every 2 days. Tumor volume was calculated by the following formula: $width^2 \times length/2$. After the treatment course, the mice were sacrificed at random and the tumors were removed for histopathological analysis including H&E staining and Masson staining. H&E staining of bilateral lungs was utilized to visualize tumor lung metastases. Immunohistochemical staining (for USP8) of the tumors was subsequently performed. Immunofluorescence staining ($LC3^+$ and $CD8^+$ cells) was also detected after treatment.

Combination therapy

After the tumors grew to ~100 mm^3, the Balb/c mice were randomized into four groups (n = 5 per group) with the following treatments: (1) control, PBS, (2) intravenous injection of 100 μg/ml of α-PD-L1, (3) intratumoral injection of 5.0 mg/kg of MnS_x, and (4) intravenous injection of 0.1 mg/ml of α- PDL-1 and intratumoral injection of 5.0 mg/kg MnS_x. MnS_x was injected on days 0, 1, and 2, while α-PD-L1 (BioXCell, USA) was injected on days 2, 4, and 6. Tumor volume and body weight were recorded every 2 days. Tumor volume was calculated by the following formula: $width^2 \times length/2$. After the treatment course, the mice were sacrificed at random, and the tumors were removed for histopathological analysis including H&E staining and Masson staining. Immunofluorescence staining (TBKI and PD-L1) was also performed after treatment.

In vivo toxicity evaluation

For toxicity evaluation, healthy Balb/c mice were divided into 3 groups (n = 5) for short-term and long-term biosafety of MnS_x. (1) control (non-treatment), (2) 1 day (raising for 1 day after intravenous injection of MnS_x, 10 mg/kg), and (3) 28 days (raising for 14 days after intravenous injection of MnS_x, 10 mg/kg). Then these mice were sacrificed at the same time and the main organs (heart, liver, spleen, lung, and kidney) were collected in 4% paraformaldehyde solution for tissue fixation. Blood and serum were collected for routine blood examination and serum biochemistry analysis.

Statistics

All quantitative experiments were performed in triplicate unless otherwise indicated. The data are presented as the mean ± standard deviation (SD). Statistical differences in survival were measured by the log-rank test. The significance was expressed with *$p < 0.05$, **$p < 0.01$, and ***$p < 0.001$.

2. Supporting Figures

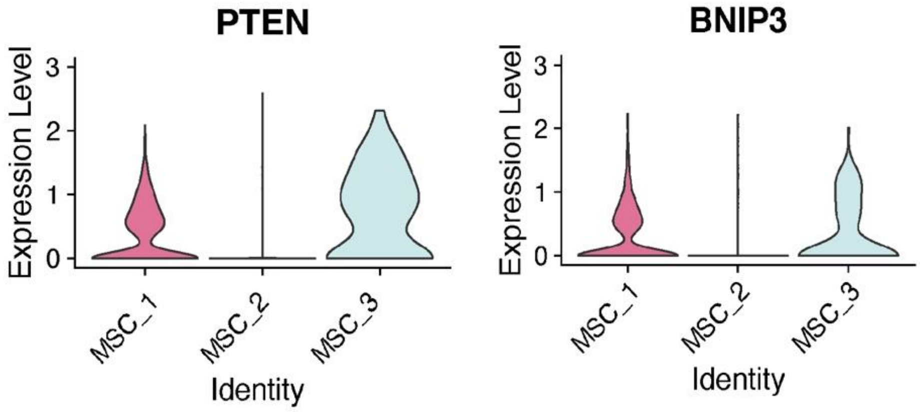

Figure S1. Violin plots showing normalized expression levels of autophagy-associated genes for each cell cluster marker.

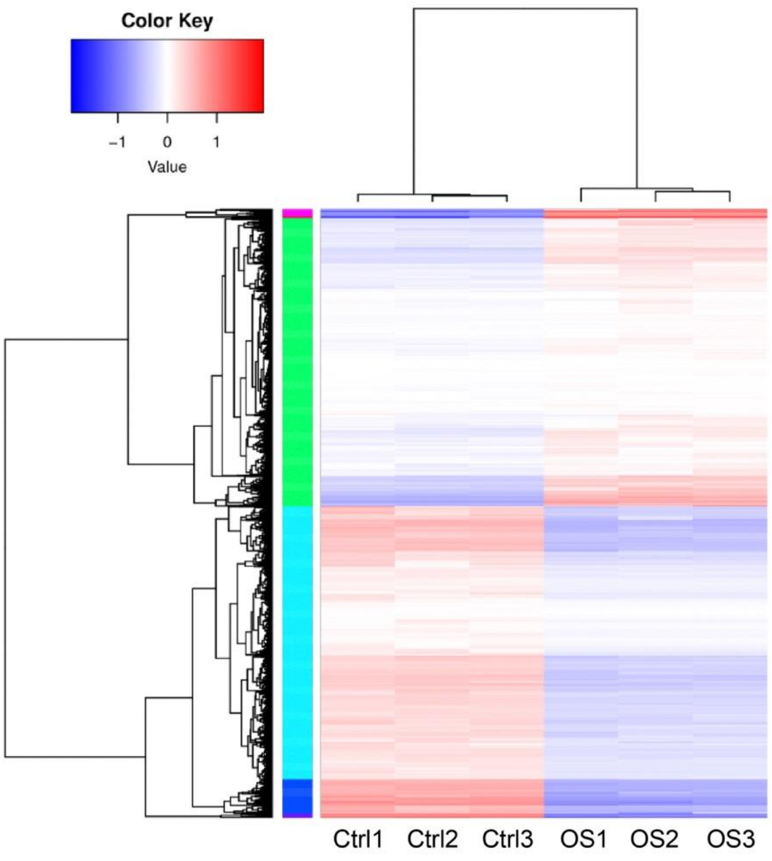

Figure S2. The heatmap of differentially expressed genes.

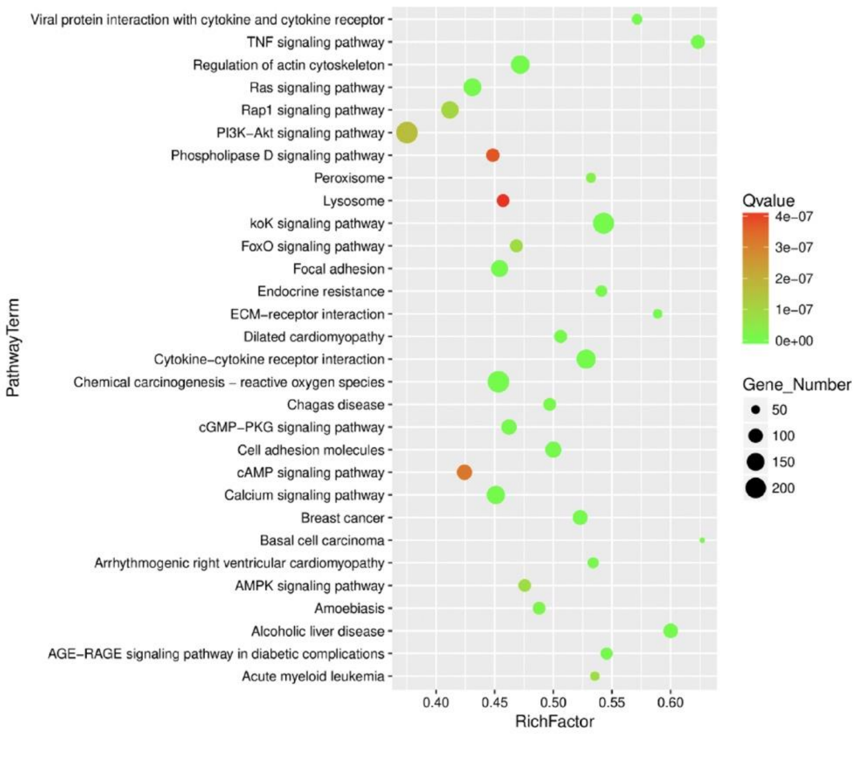

Figure S3. KEGG biological process enrichment analysis of DEGs.

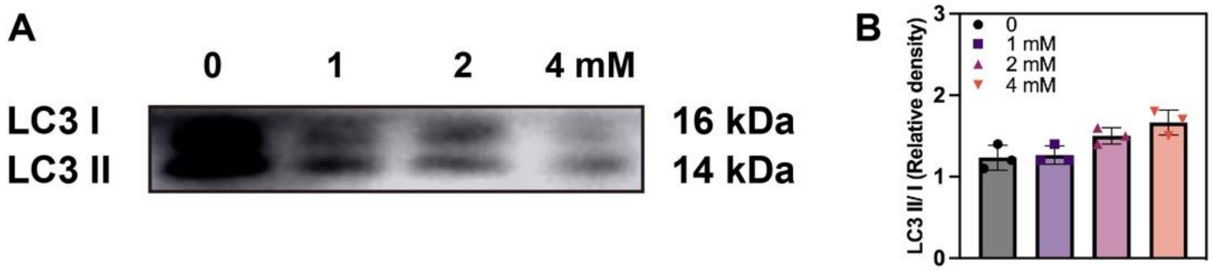

Figure S4. LC3 protein expression after treatment with different concentrations of NaHS.

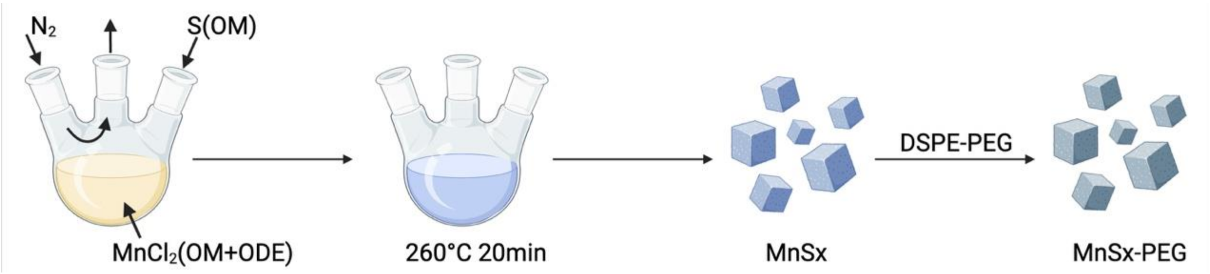

Figure S5. Schematic diagram of the synthesis and modification of MnS_x NPs.

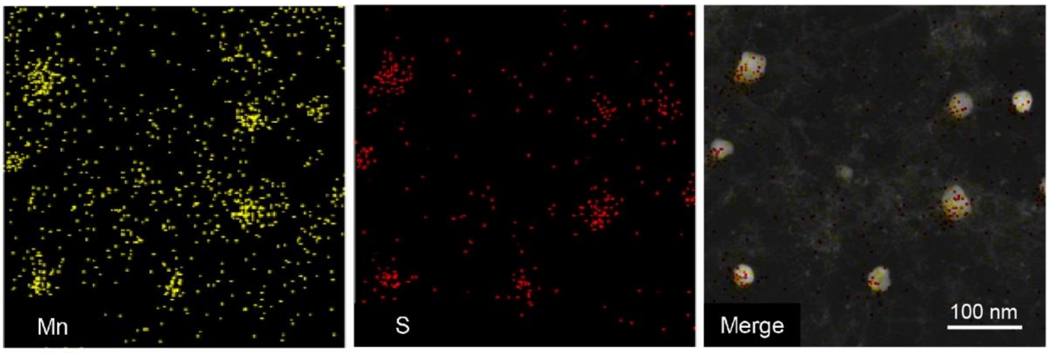

Figure S6. Element mapping images of MnS_x (scale bar = 100 nm).

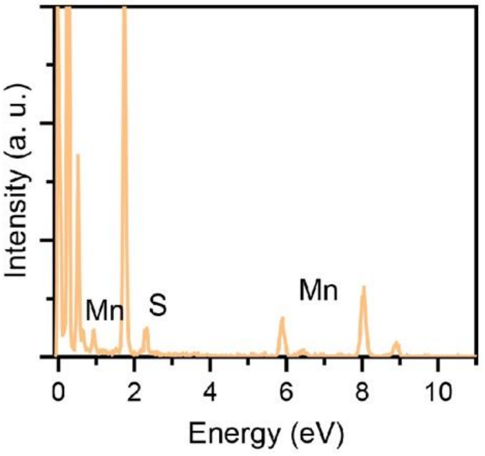

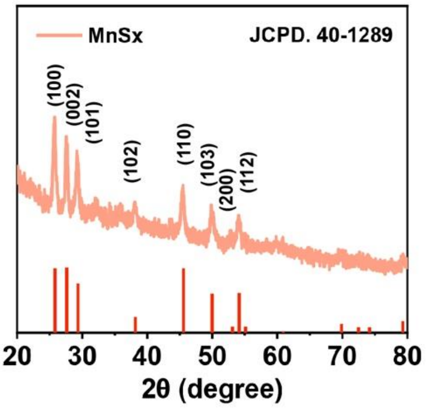

Figure S7. EDS spectrum of the MnS_x NPs.
Figure S8. XRD analysis of MnS_x.

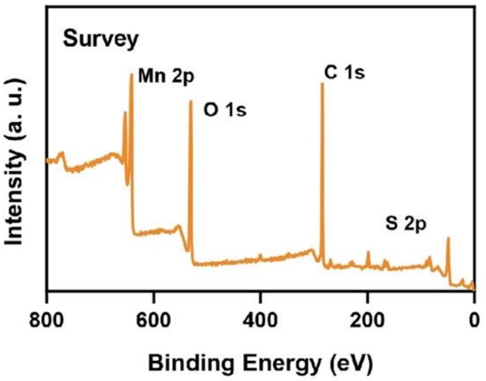

Figure S9. XPS spectrum of MnS_x NPs after the release of H_2S gas.

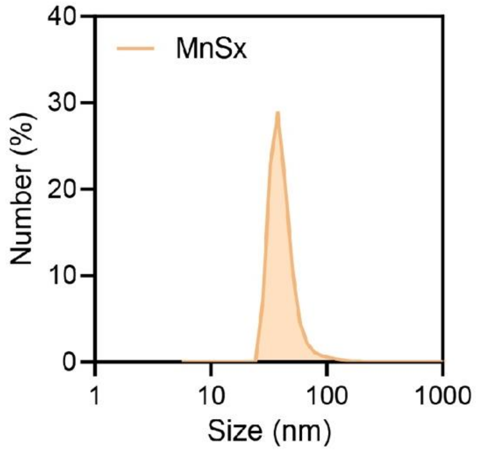

Figure S10. Hydrodynamic diameter of MnS_x.

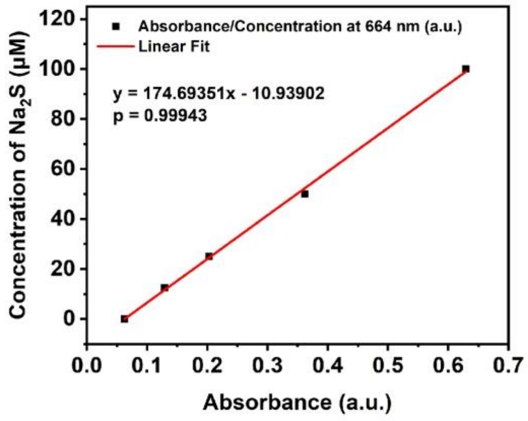

Figure S11. The linear relationship between NaHS concentration and absorption intensity.

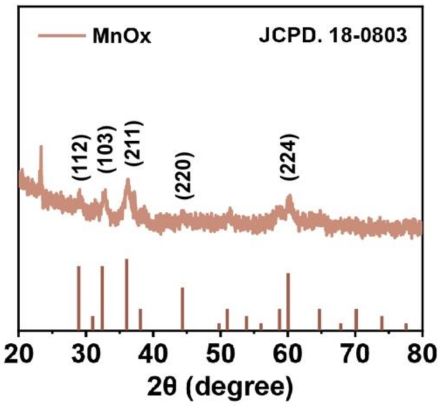

Figure S12. XRD pattern of MnOx.

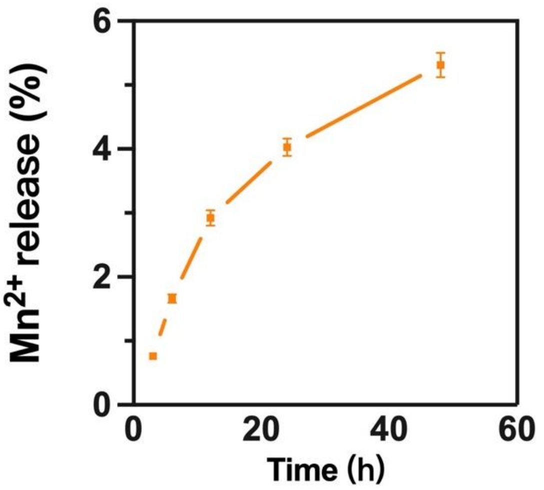

Figure S13. The proportions of Mn^{2+} release from MnS_x at different incubation time.

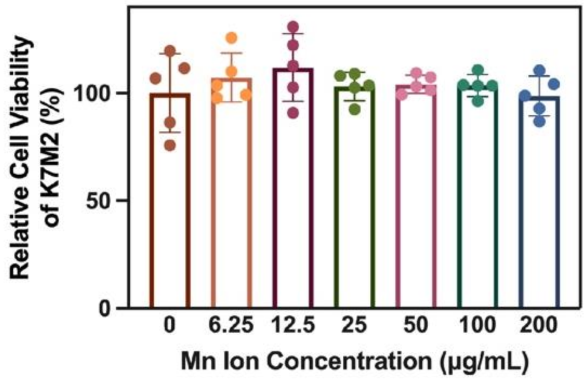

Figure S14. Relative viability of K7M2 cells after treatment with different concentrations of Mn^{2+}.

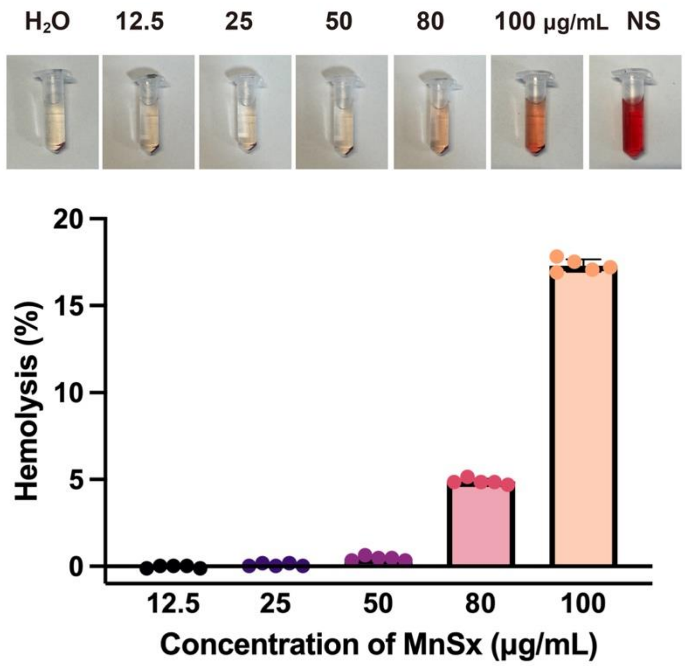

Figure S15. Hemolysis tests of MnS_x with different concentrations.

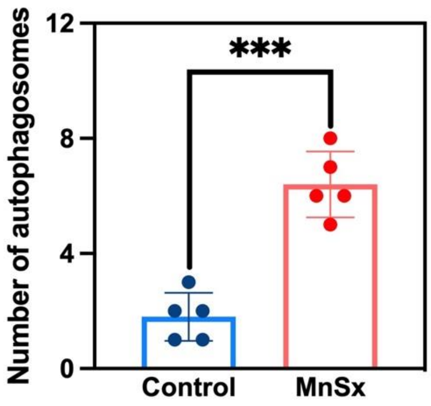

Figure S16. TEM analysis of the number of autophagosomes in K7M2 cells after treatment with $MnS_{x.}$

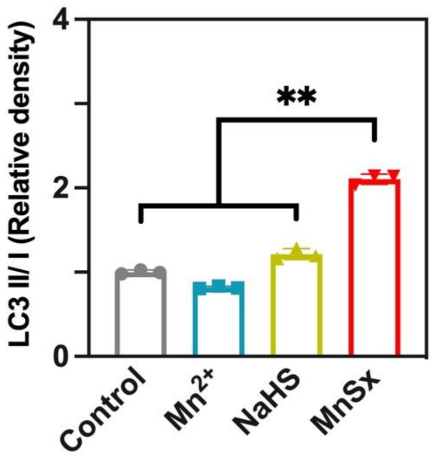

Figure S17. Quantitative analysis of LC3 protein expression after different treatments.

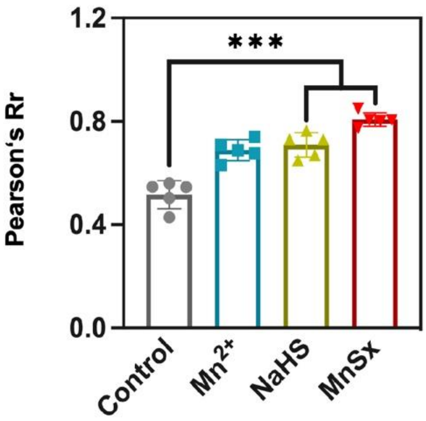

Figure S18. Pearson's index for co-localisation of mitochondria with lysosomes was calculated by ImageJ.

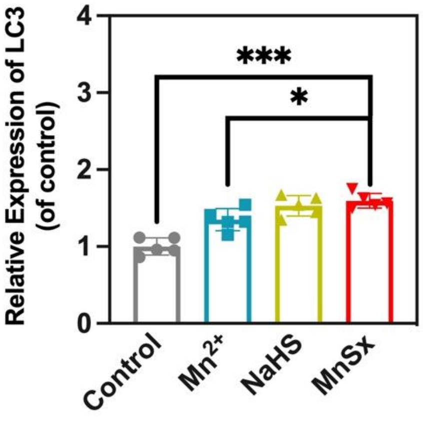

Figure S19. Relative expression of LC3 in K7M2 cells after different treatments.

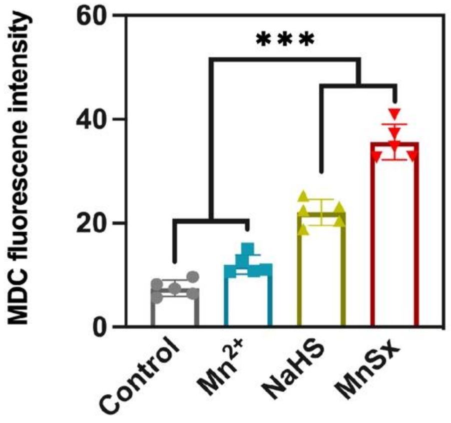

Figure S20. Quantitative analytical assay of autophagosomes for the MDC assay.

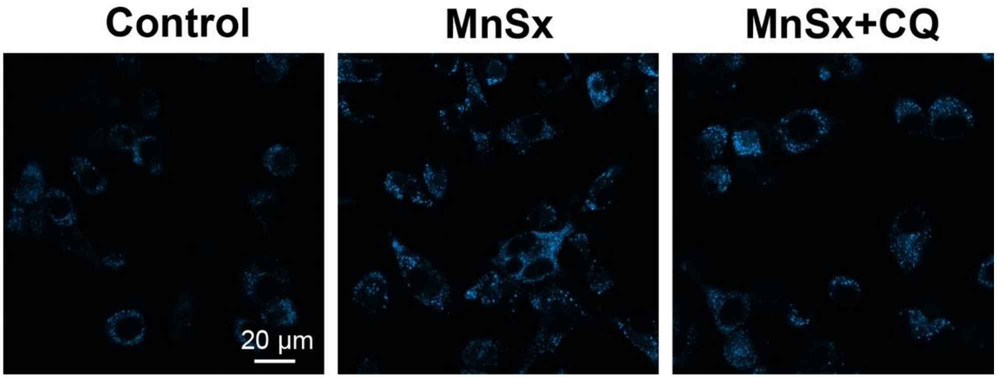

Figure S21. Detection of autophagosomes (blue) in K7M2 cells by the MDC test.

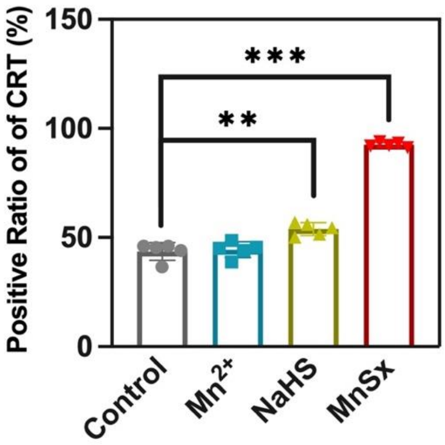

Figure S22. Quantitative analysis of the expression of CRT in K7M2 cells was detected by flow cytometry.

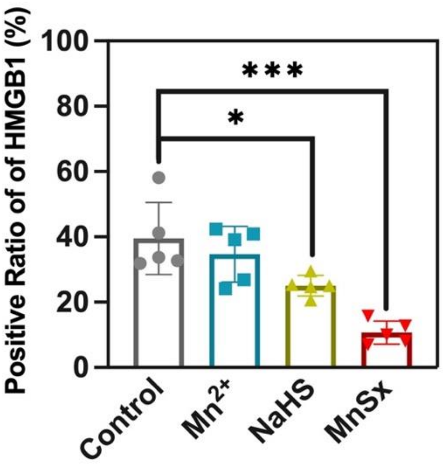

Figure S23. Quantitative analysis of the expression of HMGB1 in K7M2 cells detected by flow cytometry.

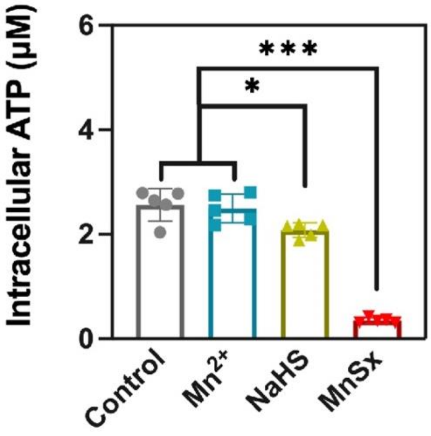

Figure S24. Quantitative analysis of ATP release.

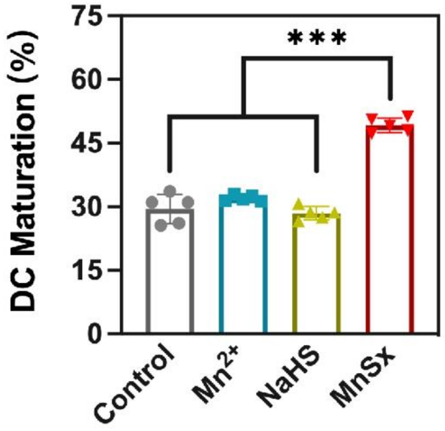

Figure S25. Quantitative analysis of DC maturation.

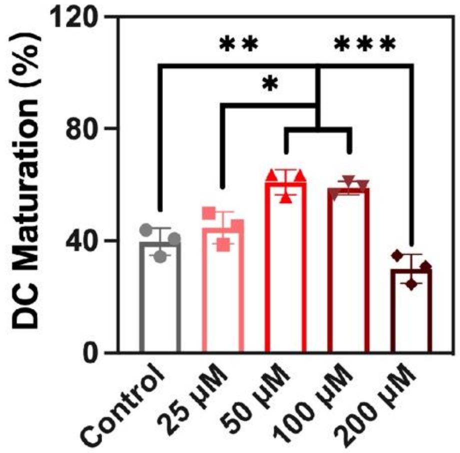

Figure S26. The percentage of DC maturation after treatment with different concentrations of MnS_x.

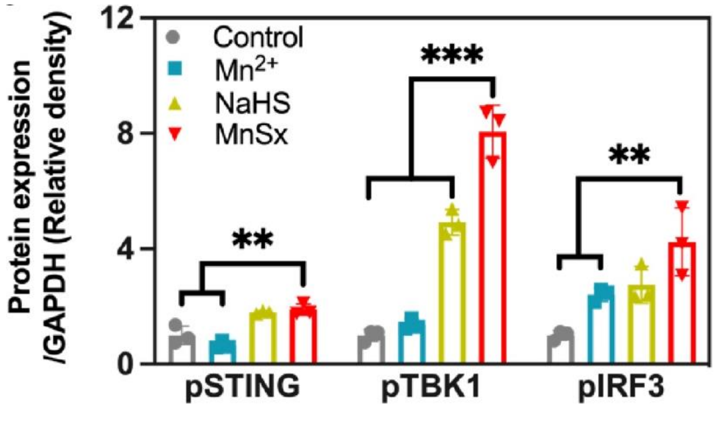

Figure S27. Quantitative analysis of Sting-related protein expression.

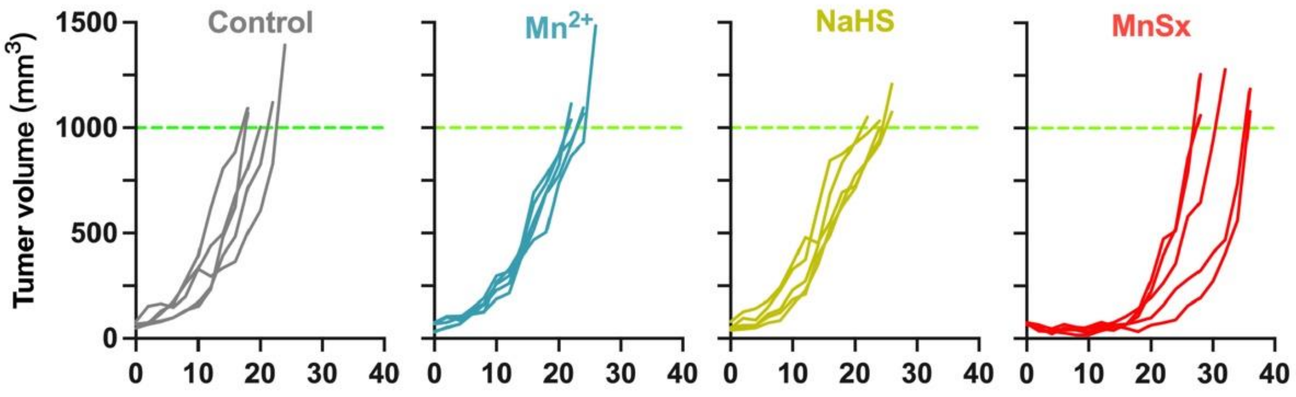

Figure S28. Tumor size of mice after various treatments.

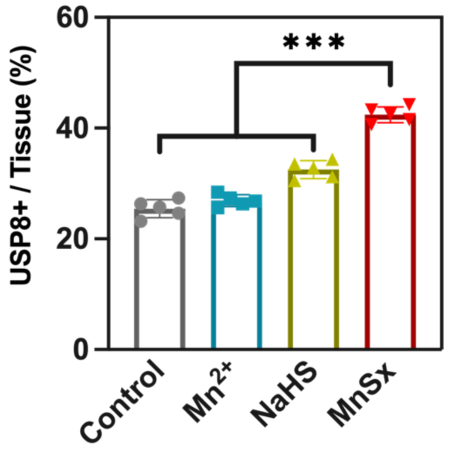

Figure S29. Quantitative analysis of the percentage area of immunohistochemical staining for USP8.

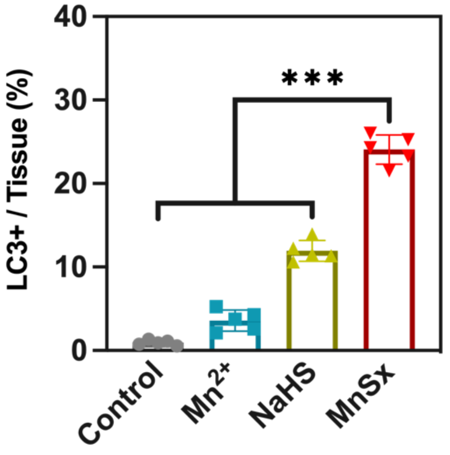

Figure S30. Quantitative analysis of the percentage area of immunofluorescence staining for LC3.

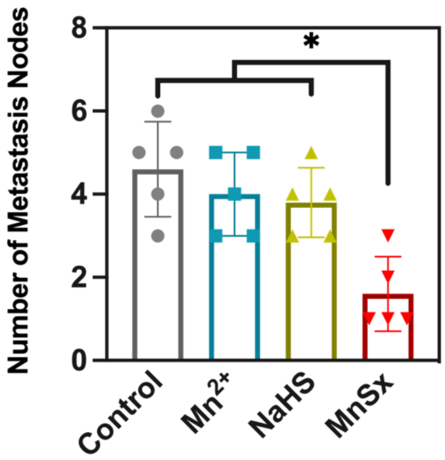

Figure S31. Quantitative analysis of lung metastases

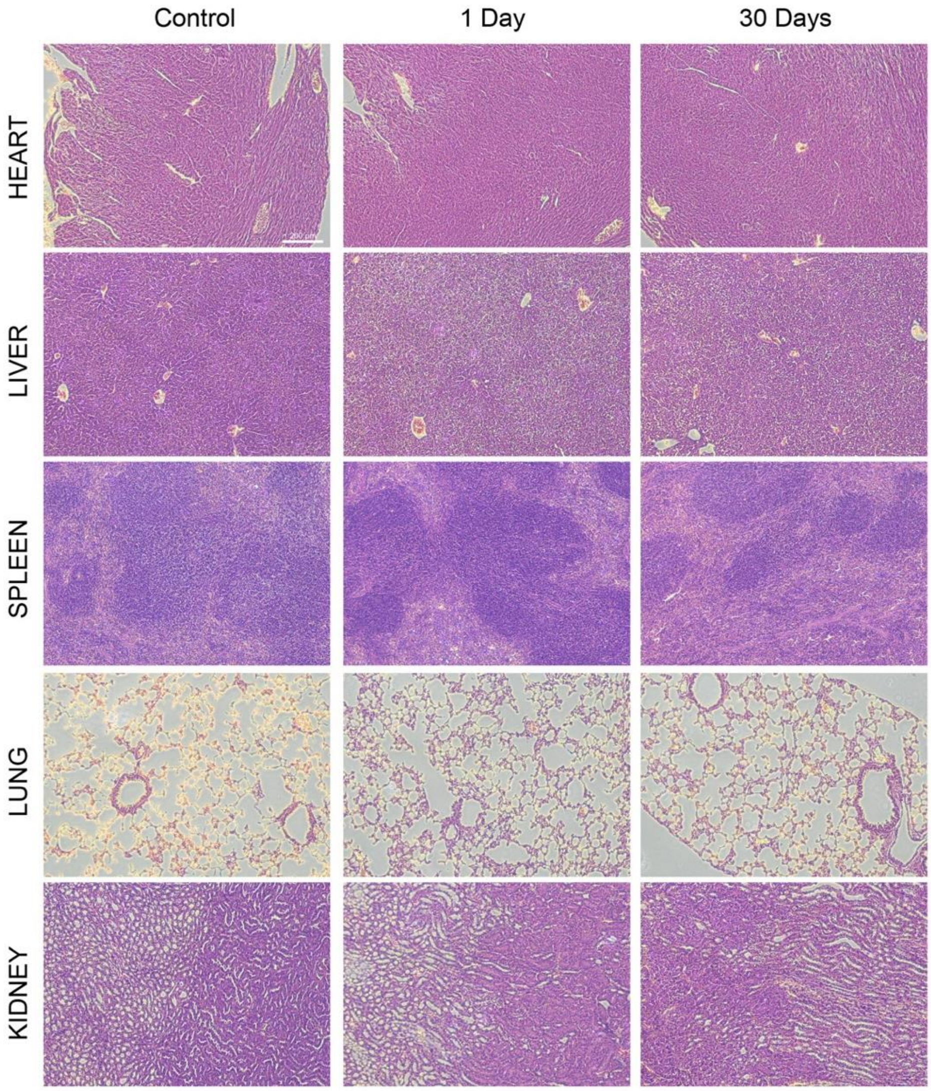

Figure S32. Toxicity of MnS_x. H&E images of major organs from mice before and post i.v. injection with MnS_x (10 mg/kg) at 1 d and 30 d, respectively. Scale bar: 200 μm.

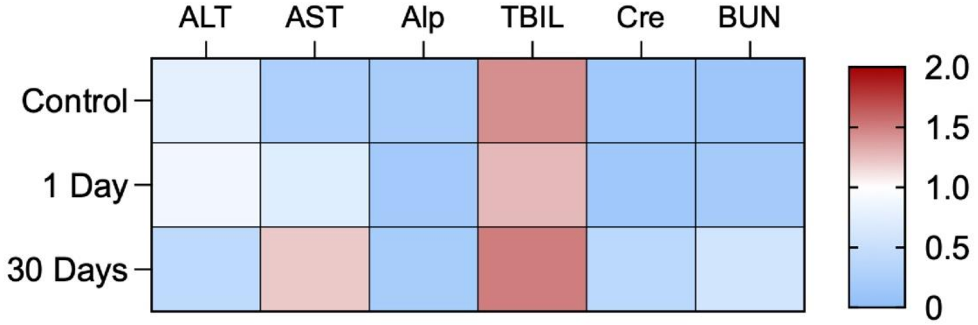

Figure S33. Toxicity of MnS_x. Blood biochemistry test from mice before and post i.v. injection with MnS_x (10 mg/kg) at 1 d and 30 d, respectively.

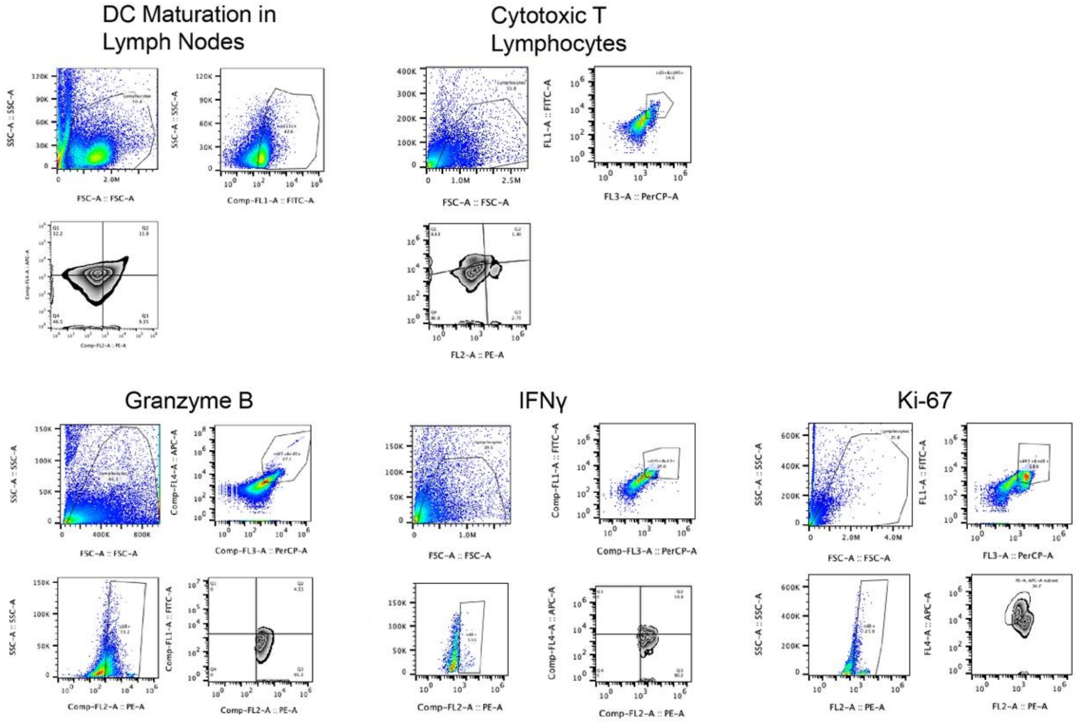

Figure S34. Gate selection strategies for single cells in flow cytometry.

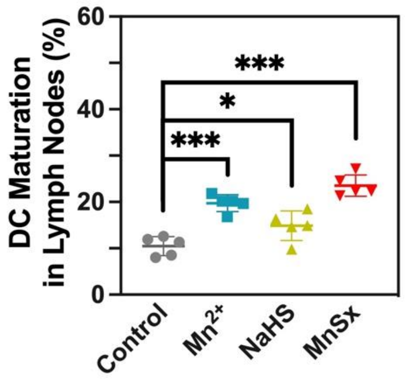

Figure S35. Representative flow cytometric analysis of DC maturation ($CD80^+$ $CD86^+$) in lymph nodes, gating on $CD11c^+$ cells.

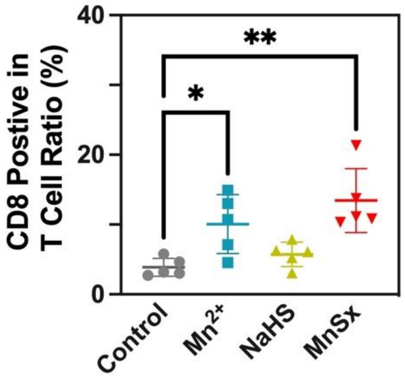

Figure S36. $CD8^+$ T cells among $CD3^+$ $CD45^+$ T cells in the tumors.

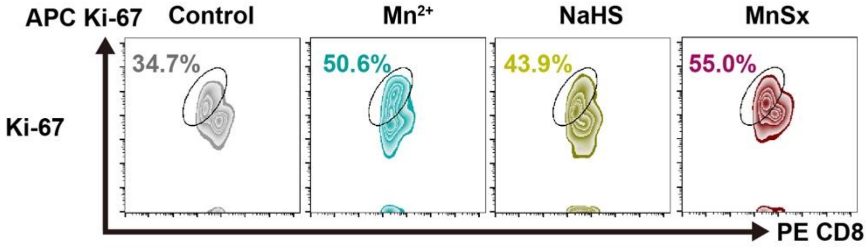

Figure S37. Ki-67^+ $CD8^+$ T cells among $CD3^+$ $CD45^+$ T cells in tumors.

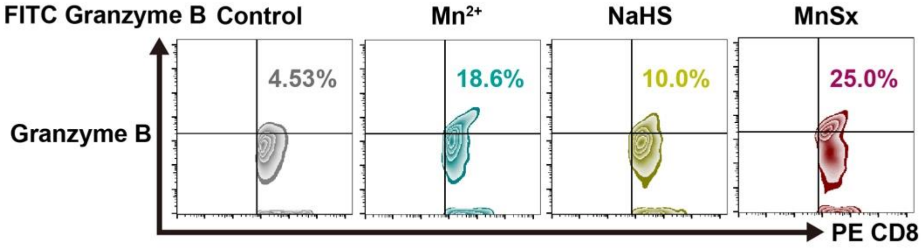

Figure S38. Granzyme$^+$ CD8$^+$ T cells among CD3$^+$ CD45$^+$ T cells in tumors.

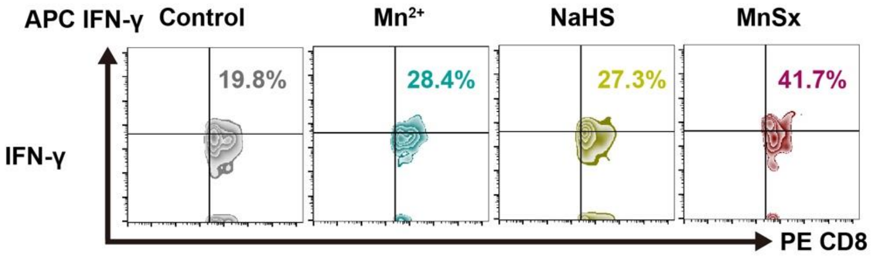

Figure S39. IFN-γ$^+$ CD8$^+$ T cells among CD3$^+$ CD45$^+$ T cells in tumors.

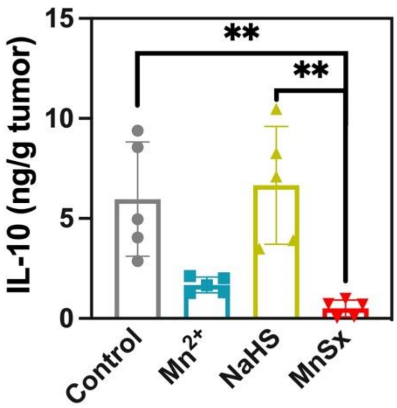

Figure S40. Detection of the cytokines IL-10 in tumors.

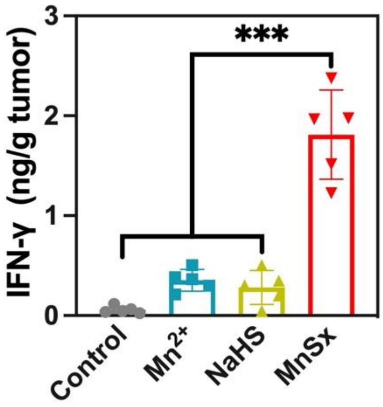

Figure S41. Detection of the cytokines IFN-γ in tumors.

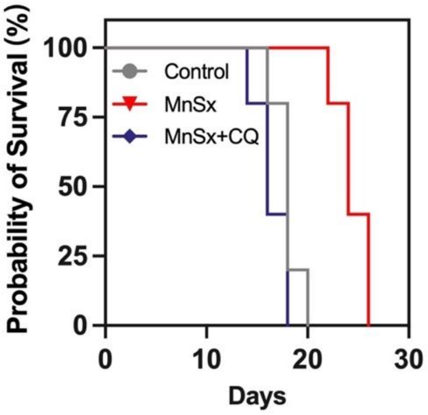

Figure S42. Survival curve of mice after the treatments.

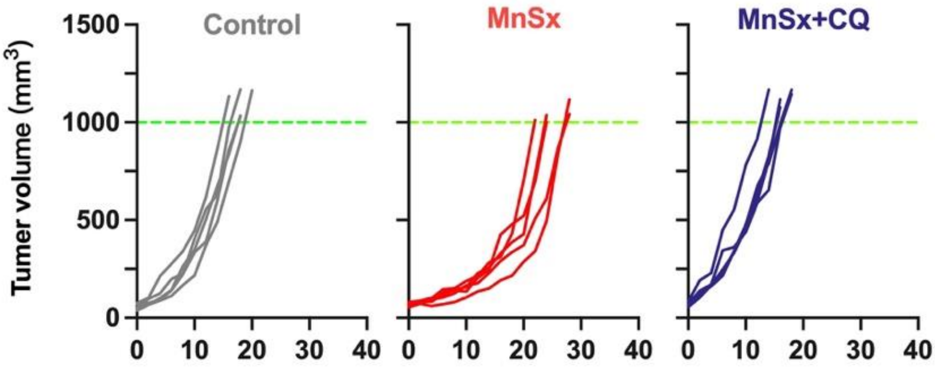

Figure S43. Tumor size of mice after various treatments.

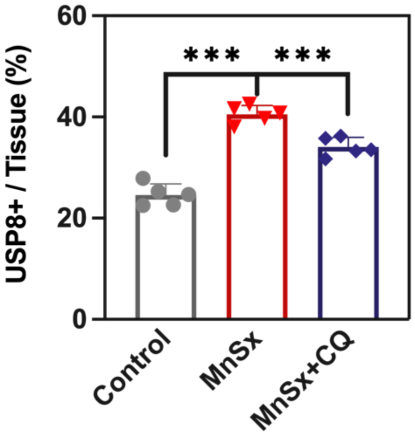

Figure S44. Quantitative analysis of the percentage area of immunohistochemical staining for USP8.

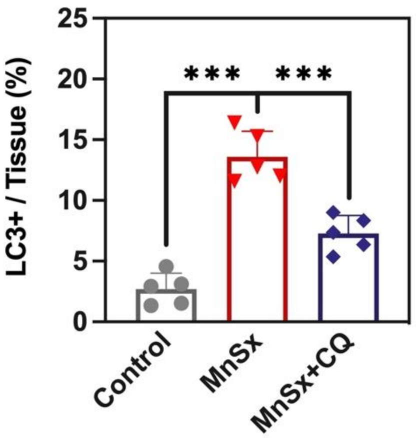

Figure S45. Quantitative analysis of the percentage area of immunofluorescence staining for LC3.

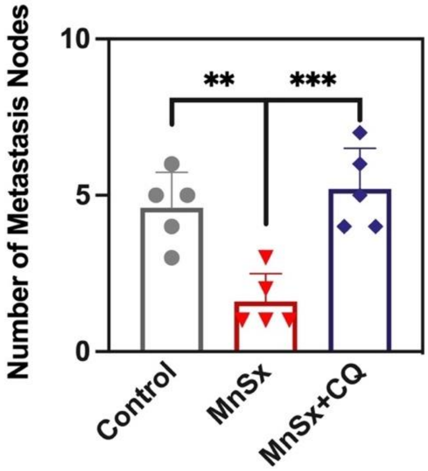

Figure S46. Quantitative analysis of lung metastases after various treatments.

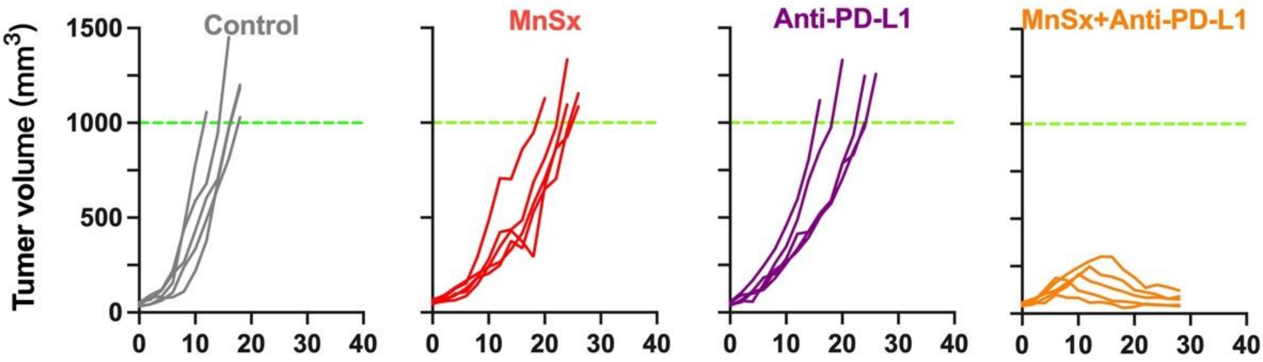

Figure S47. Tumor size of mice after various treatments.

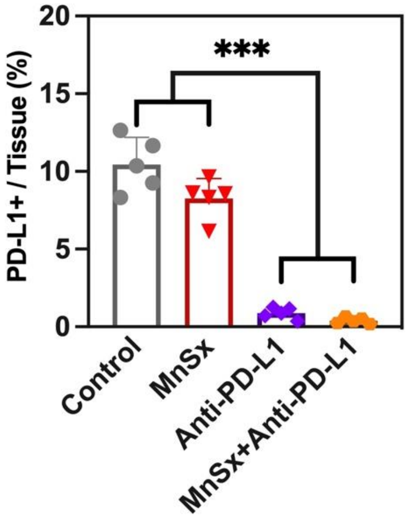

Figure S48. Quantitative analysis of the percentage area of immunofluorescence staining for PD-L1.

Table1. The primer sequence of each gene.

Genes	5’- Sense primer sequence -3’	5’- Antisense primer sequence -3’
Map1lc3b	GATAATCAGACGGCGCTT	ACTTCGGAGATGGGAGTG
Atg4d	AGGGGACAAACCCGTATCC	CCATACTTGACGTTGTTCCAGG
Atg9a	CCGAGGGGAGCAAATCACC	TAGTCCACACAGCTAACCAGG
Atg13	CCAGGCTCGACTTGGAGAAAA	AGATTTCCACACACATAGATCGC

Atg14	GAGGGCCTTTACGTGGCTG	AATAGACGAAATCACCGCTCTG
GAPDH	AGGTCGGTG TGAACGGATTTG	GGGGTCGTTGATGGCAACA
